# Efficiency of feed and energy use in primiparous and multiparous dairy cows fed contrasting dietary protein concentrations across lactation

**DOI:** 10.1016/j.animal.2025.101426

**Published:** 2025-03

**Authors:** S. Ormston, T. Yan, X. Chen, A.W. Gordon, K. Theodoridou, S. Huws, S. Stergiadis

**Affiliations:** a Department of Animal Sciences, School of Agriculture, Policy and Development, University of Reading, PO Box 237, Earley Gate, Reading, RG6 6EU, United Kingdom; b Agri-Food and Biosciences Institute, Hillsborough, Co. Down, BT26 6DR, United Kingdom; c Agri-Food and Biosciences Institute, Statistical Services Branch, Newforge Lane, Belfast, Co. Antrim, BT9 5PX, United Kingdom; d Institute for Global Food Security, Queen’s University Belfast, Belfast, BT9 5DL, United Kingdom

**Keywords:** CP, Energy use efficiency, Feed efficiency, Methane emissions, Parity

## Abstract

Reducing dietary CP concentration can reduce feeding costs and N excretion in dairy production but may negatively impact productivity and efficiency. This study investigated the impact of reduced dietary CP concentration, across early, mid− and late−lactation stages in primiparous and multiparous lactating cows, on productivity, feed and energy use efficiency and CH_4_ emission parameters. Twenty-four Holstein-Friesian (12 primiparous, 12 multiparous) cows were allocated to three experimental total mixed rations containing 12.2, 15.1 or 18.1% CP (**LCP**, **MCP** and **HCP,** respectively; DM basis), in a continuous study across lactation (days 1–305). Digestibility and gas exchanges were measured in metabolism units and indirect open-circuit respiration calorimeter chambers during early, mid− and late−lactation. Data were analysed using a linear mixed model, with repeated measures, with CP concentration, stage of lactation, parity and their interactions as fixed effects, and a random effect of cow fitted as the subject and stage of lactation as the repeated measure. When compared with LCP treatment, MCP and HCP had higher DM intake (**DMI**) (+1.9 and +3.0 kg/d), milk yield (+5.5 and +7.7 kg/d), energy corrected milk yield (**ECMY**) (+5.0 and +7.1 kg/d), and feed efficiency (ECMY/DMI, +0.13 and +0.15 kg/kg; milk solids/DMI, +16 and +20 g/kg; milk energy output (E_L_)/DMI, +0.40 and 0.46 MJ/kg). Digestible energy intake (**DEI**)/gross energy intake (**GEI**), metabolisable energy intake (**MEI**)/GEI, MEI/DEI and milk energy output adjusted for zero energy balance (E_L(0)_)/MEI were higher in HCP (+0.02, +0.03, +0.02, and +0.06 MJ/MJ) than LCP while there were no differences between LCP and MCP for DEI/GEI, or between MCP and HCP for MEI/DEI and E_L(0)_/MEI. Methane production per digestible DMI and ECMY were lower for MCP (−3.2 and −3.1 g/kg) and HCP (−3.8 and −3.4 g/kg), when compared with LCP. Methane energy per GEI, DEI and MEI were lower for the MCP (−0.007, −0.011 and −0.014 MJ/MJ) and HCP (−0.007, −0.014 and −0.017 MJ/MJ) than LCP. The significant interaction between CP concentration and stage of lactation on milk yield and ECMY showed that treatment differences (increased values in MCP or HCP vs LCP diets) reduced as lactation progressed. A diet containing 15.1% CP (DM basis) may be sufficient to maintain milk production and feed efficiency while reducing CH_4_ yield and intensity. However, regression analysis suggests that productivity may increase further between 15 and 18% CP (DM basis) but the response to dietary CP may depend on the stage of lactation.

## Highlights

• The impact of reducing diet CP on feed efficiency and methane emissions was assessed.
• Productivity and efficiency were reduced when diets had 12.2% CP.
• Feeding 15.1% than 18.1% CP did not affect feed efficiency and methane emissions.
• Feeding 17% CP resulted in optimum milk yield.
• Feeding 16% CP resulted in optimum feed efficiency.

## Implications

Reducing dietary CP can reduce nitrogen excretion in manure but may also reduce milk yield; while simultaneous assessments on energy use efficiency and methane production parameters are scarce. This study found that a diet with a CP concentration between 15 and 18% (DM basis) may provide sufficient protein for primiparous and multiparous lactating dairy cows to maintain milk production and feeding efficiency whilst reducing CH_4_ yield and intensity (when compared with lower protein diets (12.2% of DM)). The optimal dietary CP content for milk production was 17%, although an optimum CP concentration within this range (15–18%) may depend upon the lactation stage.

## Introduction

Utilisation of dietary protein in the dairy cow is relatively inefficient, with approximately 25–30% being stored and captured and the remainder being excreted as nitrogen (N) in faeces and urine (Broderick, 2003). Subsequent conversion of manure N to ammonia and nitrous oxide via microbial fermentation contributes to greenhouse gas emissions and leeching of nitrates from soil results in eutrophication of ground water (Tamminga, 2003, Hoekstra et al., 2020). There is growing pressure on the dairy industry to reduce its environmental impact (Hoekstra et al., 2020), with one pathway being to lower dietary CP. However, reductions in dietary CP often result in subsequent reductions in milk yield, especially when dietary CP protein reaches relatively low concentration such as 11.8–15.1% DM (Wu and Satter, 2000, Broderick, 2003, Olmos Colmenero and Broderick, 2006, Barros et al., 2017, Liu and VandeHaar, 2020). In contrast, other studies suggest that increasing dietary CP beyond 14.4–16.7% DM may not result in significant increases in milk production (Broderick, 2003, Olmos Colmenero and Broderick, 2006, Barros et al., 2017). Therefore, it seems that the optimal CP concentration may differ between investigations and warrants further examination. Increased milk yield in response to increasing CP concentration (Olmos Colmenero and Broderick, 2006, Broderick et al., 2009, Barros et al., 2017, Liu and VandeHaar, 2020, Letelier et al., 2022) has primarily been linked to increases in DM intake (**DMI**), which may also affect feed efficiency (Barros et al., 2017, Liu and VandeHaar, 2020).

Considering the growing world population (FAO, 2017), the increasing demand for animal products (Huws et al., 2018) and the subsequent competition for natural resources (FAO, 2017), improvements in feed efficiency are imperative to ensure the profitability of dairy systems (Broderick, 2003). This is particularly pertinent when considering the recognised high cost of protein in animal diets (Cabrita et al., 2007). Furthermore, ruminant livestock represent a risk to climate change, with agriculture contributing approximately 47% towards overall CH_4_ emissions in the UK (DEFRA, 2021a), mainly from enteric fermentation of ruminant animals (DEFRA, 2021b). Eradicating CH_4_ production completely is not possible, and mitigation strategies should also consider productivity and feed efficiency (Knapp et al., 2014). Not only is CH_4_ cause for concern from an environmental perspective, but it represents a loss of 3.7–10.1% of gross energy intake (**GEI**) in dairy cows (Yan et al., 2000). Thus, any reduction in CH_4_ production could increase the availability of metabolisable energy (**ME**) and improve energy use efficiency (energy output in milk/gross energy intake (Phuong et al., 2013, Hynes et al., 2016)). Despite the relationship between energy use efficiency and CH_4_ production parameters (Yan et al., 2010), previous studies have found no difference in energy use efficiency in response to differing diet CP concentrations (Hynes et al., 2016).

Although the effect of dietary factors such as feed intake, diet quality, dietary ingredients and chemical composition on CH_4_ or energy use efficiency has been widely studied (Agnew and Yan, 2000, Yan et al., 2010, Muñoz et al., 2015, Hynes et al., 2016), few studies investigate the impact of dietary CP concentration, particularly throughout lactation. Furthermore, CP concentrations investigated are relatively high and represent the range already typically fed to dairy cows (16–18% CP of DM (Webster, 2020)), rather than a reduced CP supply which is recently under increased interest due to financial and environmental reasons (requirement to reduce production costs and environmental footprint of livestock (Hoekstra et al., 2020)).

Considering the high cost of protein and the poor nitrogen use efficiency (less than 25% (Chen et al., 2020)), and the growing pressure for dairies to reduce dietary CP in cows’ diets, it is imperative to identify an optimal dietary CP concentration that does not negatively impact productivity and feeding efficiency, but also does not increase CH_4_ production and intensity. No study exists that investigates the impact of reduced CP intake on productivity, feed efficiency, energy use efficiency and CH_4_ production parameters, simultaneously under the same study, across lactation whilst considering animal factors (parity and stage of lactation). Therefore, the aim of this study was to (i) investigate the effect of diet CP concentration, stage of lactation and parity and their potential interactions on productivity, feed efficiency, energy use efficiency and CH_4_ production parameters, and (ii) develop correlations between diet CP concentration and measured parameters which were significantly affected by diet CP concentration, including data from different lactation stages. We hypothesise that diets with contrasting CP concentrations will affect productivity, feed efficiency, energy use efficiency and CH_4_ production parameters; and that the extent of these differences would vary between the different stages of lactation.

## Material and methods

### Experimental design, animals and diet

All procedures adopted in the present experiments were approved by the Ethical Review Committee of the Agri-Food and Biosciences Institute (Hillsborough, United Kingdom) under the Project Licence of 2587b and were in accordance with the UK Animal Scientific Procedures Act (1986).

Data used in the present study were collated from a metabolism study with 24 autumn-calving lactating Holstein-Friesian dairy cows (12 primiparous and 12 multiparous). These cows were a subset of cows from a whole lactation study, described in full in the supplementary material (Law et al., 2009). The whole lactation study allocated 90 Holstein-Friesian dairy cows (45 primiparous and 45 multiparous, with a mean parity of 3.1) to total mixed rations containing one of 3 dietary treatments; Low (**LCP**), Medium (**MCP**) or High (**HCP**) CP concentrations, with target CP concentrations of 120, 150 and 180 g/kg DM). All cows remained on their allocated diets from calving until day 150 of lactation. From day 151–305 of lactation, half of the animals on each treatment remained on their original treatment diets, while the other half of cows changed to a different CP treatment. Before the commencement of the main production study (Law et al., 2009), the 90 cows used were blocked into 30 groups, balanced within each group for parity, calving date, live weight and milk yield. The three cows within each group were then randomly allocated to the low, medium and high CP diet treatments. From the cows on their original treatments during the whole lactation, the same eight animals from each CP treatment (four primiparous and four multiparous) were selected from the same blocked groups for the present metabolism study. These 24 cows were transferred to metabolism units at days in milk of 70 – 90 (early), 150 – 170 (mid) and 230 – 250 (late) (details described latter). In the present metabolism study, days pregnant for early, mid− and late−lactation were, 0 ± 0, 39.1 ± 42.0, 92.1 ± 73.5 for LCP, 1.6 ± 3.1, 38.3 ± 42.1, 115.8 ± 92.5 for MCP, and 0 ± 0, 26.1 ± 44.0, 71.6 ± 85.6 for HCP. All three diets consisted of 270 g/kg DM of grass silage (predominantly perennial ryegrass), 180 g/kg DM of maize silage and 550 g/kg DM of concentrate feed. Actual CP concentrations for the 24 cows in the current study were 122, 151 and 181 g/kg DM for LCP, MCP, and HCP, respectively. The different dietary CP concentrations were achieved through weekly manipulation of input ratios of two concentrate feeds which contained either low or high CP concentrations (117 vs 229 g/kg DM). Details of dietary ingredients and chemical compositions of treatment diets are presented in Table 1. Details of the ingredients and chemical composition of the two concentrate feeds used are presented by Law et al. (2009). Briefly, the high CP concentrate included (g/kg DM) barley (140), wheat (140), unmolassed sugar beet pulp (94), citrus pulp (94), maize gluten feed (100), distillers maize grain (100), soybean meal (165), rape meal (100), tallow (14), minerals and vitamins (29) and molasses (24). The low CP concentrate included barley (240), wheat (240), unmolassed sugar beet pulp (163), citrus pulp (163), maize gluten feed (30), distillers’ maize grain (80), tallow (22), minerals and vitamins (38) and molasses (24). The ME level of high and low CP concentrate feeds were both 13.1 MJ/kg of DM but effective rumen degradable CP (ERDP) was 135 and 79 g/kg DM, digestible undegradable protein (DUP) was 74 and 29 g/kg DM and had starch concentrations of 215 and 311 g/kg DM for high and low concentrate feeds, respectively.

**Table 1:** Chemical and ingredient composition and metabolisable energy concentration of mixed diets offered to dairy cows.

	CP concentration		
Item	Low	Medium	High
Ingredient composition (g/kg DM)			
Grass silage	270	270	270
Maize silage	180	180	180
Concentrate feed^1^	550	550	550

Chemical composition (g/kg DM)			
NDF	356	358	361
ADF	188	187	186
Starch	208	190	170
CP	122	151	181
Rumen degradable protein	84	100	116
Rumen undegradable protein	38	51	65
Ash	71.0	72.0	74.0

Energy contents (MJ/kg DM)			
Gross energy	18.3	18.4	18.5
Metabolisable energy^2^	11.2	11.6	12.0

1 Target diet CP concentrations were achieved by manipulating the quantity of two different concentrate feeds (high vs low CP) containing either 229 or 117 g/kg CP DM, using the Feed into Milk programme (Thomas, 2004). Both concentrate feeds contained the same level of metabolisable energy (13.1 MJ/kg DM) but differing proportions of concentrate feed. Ingredients consisted of; barley (140 vs 240), wheat (140 vs 240), unmolassed sugar beet pulp (94 vs 163), citrus pulp (94 vs 163), maize gluten feed (100 vs 30), distillers maize grain (100 vs 80), soybean meal (Hi-Pro)(165 vs 0), rape meal (100 vs 0), tallow (14 vs 22), molasses (24 vs 24), and minerals/vitamin (29 vs 38). Low and high concentrate feeds contained 79 vs 135 g/kg DM of rumen degradable protein, respectively and 74 vs 29 g/kg DM of digestible undegradable protein. Grass silage contained ash, CP, ADF and NDF of 88, 158, 311 and 538 g/kg DM, respectively. The corresponding data for maize silage are 39, 77, 249 and 478 g/kg DM.

2 Dietary concentration of metabolisable energy was calculated using measured gross energy intake, energy outputs in faeces and urine, and actual methane energy output.

In the present metabolism study, all cows were loose housed in cubicle accommodation and had ad libitum access to total mixed ration (estimated as +5% of the previous 3-day’s feed intake) which was delivered between 1000 and 1100 h. Automatic recording feeders (Calan gate feeder) were used to measure individual animal daily feed intake (between 2 and 2.5 animals allocated per feeder). All animals had free access to water and were milked twice daily at 0500 and 1630 h. Data utilised for this investigation have previously been published, investigating the impact of varying CP concentration on milk production, nutrient digestibility and N utilisation efficiency, by (Yang et al., 2022).

### Digestibility study, calorimetric measures and sample analysis

Details of digestibility, calorimetric measures and sample analysis were outlined by Yang et al. (2022). During early (70–90 days), mid− (150–170 days) and late− (230–250 days) lactation, cows were transferred to metabolism units for 8 days with measurements of feed intake and total outputs of faeces and urine taking place during days three to eight. On day nine, cows were transferred to indirect open-circuit respiration calorimeter chambers for a further 3 days with gaseous exchange (O_2_, CO_2_ and CH_4_) measured during the final 2 days, following one day of adaptation to the chambers. Two chambers were available during the experiment, and therefore, two animals (from different treatments) were measured at each given time/measurement period. This resulted in all 24 cows being measured in 12 pairs during each period, in an interval of 3 days. Recovery rates were determined by releasing a known quantity of CO_2_ into the chambers to ensure a recovery range between 97 and 103%. The calorimetric chambers used to measure gaseous exchange in the present study were as described by Gordon et al. (1995) and Yan et al. (2000). Calibration of the chambers was carried out as follows: gases produced from individual pure analytical standard gases (CH_4_, CO_2_ and O_2_) were used to calibrate analysers using Wostoff Mixing pumps which determined the absolute range (0–500 ppm for methane) and the linearity within the range. Oxygen−free N and a known concentration of gas (span gas) were used to calibrate the analysers before each run and was checked every 6 h. Analytical grade CO_2_ and N were used to check flow measurement systems by determining the recovery of CO_2_ and depletion of O_2_ using CO_2_ and O_2_ analysers.

Cow BW and body condition score (determined by the method of Edmonson et al., 1989) were recorded on the first and last day of the digestibility measurement period, before feeding. During the measurement period, daily samples of grass and maize silage were analysed for oven DM (60 °C for 48 h). Dried samples were bulked over the week for determination of ADF, NDF and ash concentrations. Fresh silage samples were taken twice weekly when cows were loose housed and daily during digestibility and chamber measurements for determination of toluene-alcohol-corrected DM, pH, CP, ammonia-N, gross energy (**GE**), lactic acid, volatile fatty acids, ethanol and propanol concentrations as described by (Cushnahan and Gordon, 1995). Fresh concentrate samples were taken three times per week when cows were loose housed and daily during the digestibility and chamber measurements and bulked each week for analysis for oven DM (85 °C for 24 h), GE, CP, ADF, NDF and ash concentrations. For each cow, faeces and urine were separately collected using a separate patch (artificial leather) which was attached (using glue) to the back of cows. Faeces were collected using a collection tray placed at the end of the stall bed, and urine was collected into a covered container through a collection tube which was attached (using hook and loop tape) to the separate patch. A total of 300 ml 9M H_2_SO_4_ was added to the urine containers before the collection. Samples were taken and recorded daily for faeces and urine outputs (5% of total excretion) and mixed separately. Samples were then taken for analysis; fresh faecal samples for oven DM (85 °C for 48 h), GE and N, and dried samples for ADF, NDF and ash concentrations, and urine samples for GE and N concentrations. Methods for analysis of silage, concentrate, faeces and urine samples are outlined by (Cushnahan and Gordon, 1995).

Milk yield was measured daily, and milk samples were taken during the morning and afternoon milking on three consecutive days every second week during the loose housed period and daily during the digestibility and chamber measurement periods. Weekly milk samples were weighted by milk production at each milking and combined and analysed for fat and lactose concentrations using a Milkoscan Model 605 (Foss Electric, DK-3400, Hillorod, Denmark), protein concentrations were determined as Kjeldahl N × 6.38, and energy concentrations were measured in an isoperibol bomb calorimeter (Parr Instruments Co., Moline, IL). Results for milk yield have also been presented by Law et al. (2009).

### Calculations and statistical analysis

A number of energy utilisation and feed efficiency parameters were calculated. Energy−corrected milk yield (**ECMY**) was calculated based on the following equation by AFRC (1993):

$$\mathrm{ECMY}=\frac{\mathrm{Milk}\;energy\;output\left(E_{L}:MJ/d\right)}{\mathrm{Standard}\;milk\;energy\;content\left(MJ/kg\right)}$$

where E_L_ is milk energy output (MJ/d) from the following equation:

$$E_{L}=actual\;milk\;yield\;\left(during\;digestibility\;and\;chamber\;measurements\right)\times milk\;energy\;content\;\left(measured\;isoperibol\;bomb\;calorimeters\right)$$

The standard milk energy content was calculated based on the assumption of 1 kg of standard milk from Holstein cows, containing 40 g fat, 32 g CP and 48 g lactose, and the equation of Tyrrell and Reid (1965):

Standardmilkenergycontent(MJ/kg)=(40×0.0384)+(32×0.0223)+(48×0.0199)-0.108

Residual feed intake (RFI) was calculated from the following equation.

$$\mathrm{RFI}=actual\;DMI\;\left(kg/d\right)-predicted\;DM\;requirement\left(kg/d\right)$$

Where predicted DM requirement was calculated from total ME requirements for maintenance, lactation, BW change and pregnancy, predicted using models of Feed into Milk (Thomas, 2004) divided by actual dietary ME concentration measured in the present study.

$$\mathrm{Predicted}\;DM\;requirement\;\left(kg/d\right)=\frac{\mathrm{predicted}\;total\;ME\;requirement\;(MJ/d)}{\mathrm{actual}\;diet\;ME\;content\;(MJ/kg\;DM)}$$

All energy variables were directly measured, except for heat production (**HP,** MJ/d) and retained energy (energy retained or lost in daily weight change, **RE,** MJ/d).

HP was calculated based on O_2_ consumption (l/d), CO_2_ (l/d) and CH_4_ (l/d) production and urinary N excretion (g/d) using the equation of (Brouwer, 1965);

$$\mathrm{HP}=[\left(16.18\times O_{2}\right)+\left(5.16\times {\mathrm{CO}}_{2}\right)-\left(2.42\times {\mathrm{CH}}_{4}\right)-\left(5.9\times UN\right)]/1000$$

Retained energy (energy retained or lost in daily weight change) was calculated by subtracting E_L_ and HP from MEI.

Milk energy output corrected for zero energy balance (E_L(0)_) was calculated using the equation of AFRC (1993):

$$E_{L(0)}=E_{L}+a\times RE$$

where a = 0.84 if RE < 0, or a = 0.95 if RE > 0

Efficiency of utilisation of ME for milk production (K_L_) was calculated based on the equation of (Yan et al., 2006a):

$$K_{L}=E_{L(0)}/\left(MEI-{\mathrm{ME}}_{m}\right)$$

where:

ME_m_ = ME requirement for maintenance (MJ/d) (Yan et al., 2006a).

All data points were included in the analysis, resulting in 72 data points (24 cows each measured during the early, mid− and late−lactation). Data were analysed as a completely randomised blocked design using a linear mixed model with a repeated measures design (residual maximum likelihood analysis) to investigate the effect of CP concentration, stage of lactation, parity and all their interactions on productivity, energy and feed use efficiencies and CH_4_ parameters. The statistical programme used was GenStat® 18 (VSN International, 2020). The fixed effects were CP concentration (low; medium and high) (df = 2), stage of lactation (early, mid and late) (df = 2), parity (primiparous and multiparous) (df = 1), and their interactions, i.e., CP concentration × stage of lactation (df = 4), CP concentration × parity (df = 2), stage of lactation × parity (df = 2) and CP concentration × stage of lactation × parity (df = 4). Random effects were included as cow nested within the stage of lactation (cow as the subject and stage of lactation as the time factor). Correlation between time points was modelled using an autoregressive model of order one. The adequacy of the models fitted (normality and homogeneity of variance of the residuals) were assessed visually by inspection of the appropriate residual plots. As these indicated no deviation from normality, all variables were analysed untransformed. The R^2^ was calculated as the square of the correlation between the observed and fitted values. The fitted values were calculated using both fixed and random effects. When any fixed effect was significant for a measured variable (*P* < 0.05), pairwise comparisons of means were performed using Fisher’s Least Significant Difference test. In addition, linear and quadratic effects were also included in the statistical model for the main fixed effect of diet CP concentration and its interactions with parity and stage of lactation (CP concentration, CP concentration × stage of lactation, CP concentration × parity and CP concentration × stage of lactation × parity) as follows:

$$Y_{\mathrm{ij}}=M+D_{r}+P_{j}+L_{s}+D.P_{\mathrm{rj}}+D{.L}_{\mathrm{rs}}+P.L_{\mathrm{js}}+D.P.L_{\mathrm{rjs}}+C.P_{\mathrm{ij}}$$

Where:

Y_ij_ – response of Cow (i) in stage of lactation (j) for variable Y.

M – overall mean.

D_r_ – main effect of CP Diet (r) for cow (i).

P_j_ – main effect of stage of lactation (j).

L_s_ – main effect of parity (s) for cow (i).

D.P_rj_ − interaction effect of CP Diet (r) for cow (i) in stage of lactation (j).

D.L_rs_ − interaction effect of CP Diet (r) with parity (s) for cow (i).

P.L_js_- interaction effect of stage of lactation (j) with parity (s) for cow (i).

D.P.L_rjs_ − interaction effect of CP Diet (r) in stage of lactation (j) with parity (s) for cow (i).

C.P_ij_ – residual effect of cow (i) in stage of lactation (j).

Descriptive statistics to generate means and SEs for presentation in Tables and Figures were carried out in Minitab® 20.2.

The second objective of the present study was to evaluate if there were any linear and/or quadratic relationships between diet CP concentration and measured parameters which were significantly affected by diet CP concentration as evaluated previously. The restricted maximum likelihood was used so that the potential random effects of cow, stage of lactation and parity could be accounted for. Both linear and quadratic regressions were tested using the following statistical model:

$$Y_{\mathrm{ij}}=a+b.CP_{\mathrm{ij}}+c.CP_{\mathrm{ij}}^{2}{+Cow}_{i}+{\mathrm{Parity}}_{r}+{\mathrm{Stage}\;of\;lactation}_{j}+e_{\mathrm{ij}}$$

Where:

Y_ij_ − response to variable Y for cow (i) in stage of lactation (j).

a − intercept.

b – regression parameter for CP concentration linear effect for cow (i) in stage of lactation (j).

c – regression parameter for CP concentration quadratic effect for cow (i) in stage of lactation (j).

Cow_i_ – random effect for cow (i).

Parity_r_ – random effect for parity (r) for cow (i).

Stage of lactation_j_ – random effect for stage of lactation (j).

e_ij_ – residual effect for cow (i) in stage of lactation (j).

If the quadratic effect was statistically significant, the quadratic relationship is presented; or else the linear relationship is presented. The statistical code used for this work in GenStat is presented in Supplementary Material S1.

## Results

### Feed intake, animal productivity and feed efficiency

#### Effect of CP concentration

Dietary CP concentration did not affect LW and body condition score, but significantly (and linearly) affected the DMI (kg/d) of all feed ingredients (Table 2). When compared to cows in the LCP group, total DMI and concentrate DMI were higher for cows in the MCP (+1.9 and 1.1 kg/d, respectively) and HCP groups (+3.0 and +1.8 kg/d, respectively) (Table 2). Intakes of grass silage and maize silage were higher in the HCP group (+0.7 and +0.5 kg/d, respectively) compared to the LCP group (Table 2). DM digestibility was higher in the HCP (+1.0 and 2.0%) compared to the MCP and LCP groups.

**Table 2:** Means ± SE and *P*-values for the effect of dietary CP concentration (low/medium/high), stage of lactation, and their interactions on feed intake, diet composition, productivity and efficiency parameters in lactating dairy cows.

	CP concentration (CP)							Stage of Lactation (SL)					Parity			
	LCP^1^	MCP^1^	HCP^1^	SE	*P*-value^2^	*P*-value^2^		Early^3^	Mid^3^	Late^3^	SE	*P*-value^2^	Primiparous	Multiparous	SE	*P*-value^2^
Item	n = 24^4^	n = 24^4^	n = 24^4^			L^5^	Q^5^	n = 24^4^	n = 24^4^	n = 24^4^			n = 36^4^	n = 36^4^		
Feed intake and liveweight																
Liveweight (kg)	554	536	536	14.0	0.419	0.241	0.559	508^c^	535^b^	584^a^	13.2	<0.001	500	584	9.7	<0.001
BCS	2.52	2.43	2.44	0.056	0.433	0.281	0.479	2.34^b^	2.43^b^	2.61^a^	0.052	<0.001	2.38	2.54	0.045	0.030
Total DMI (kg/d)	16.5^b^	18.4^a^	19.5^a^	0.43	<0.001	<0.001	0.337	18.4^a^	18.4^a^	17.6^b^	0.49	0.031	16.7	19.6	0.33	<0.001
Grass silage DMI (kg/d)	4.19^b^	4.67^ab^	4.89^a^	0.140	<0.001	<0.001	0.299	4.55^ab^	4.78^a^	4.45^b^	0.156	0.048	4.14	5.03	0.098	<0.001
Maize silage DMI (kg/d)	2.82^b^	3.13^ab^	3.35^a^	0.086	<0.001	<0.001	0.587	3.26^a^	2.97^b^	3.06^ab^	0.092	0.001	2.84	3.36	0.066	<0.001
Concentrate DMI (kg/d)	9.49^b^	10.6^a^	11.3^a^	0.25	<0.001	<0.001	0.418	10.7^a^	10.7^a^	10.1^b^	0.28	0.020	9.71	11.2	0.20	<0.001
CP intake (g/d)	201	278	351	0.1	<0.001	<0.001	0.770	281	276	273	0.1	0.400	256	297	0.1	<0.001
DM digestibility (%)	72.0^b^	73.0^b^	74.0^a^	0.50	0.020	0.008	0.368	73.0	74.0	73.0	0.50	0.173	73.0	73.0	0.40	0.945

Milk production and composition																
Milk yield (kg/d)	20.6^b^	26.1^a^	28.3^a^	1.09	<0.001	<0.001	0.226	27.9^a^	26.2^a^	20.9^b^	1.12	<0.001	23.1	26.9	0.99	0.012
ECMY (kg/d)	20.7^b^	25.7^a^	27.8^a^	0.91	<0.001	<0.001	0.107	26.8^a^	26.1^a^	20.9^b^	0.97	<0.001	22.9	26.6	0.82	<0.001
Milk fat yield (kg/d)	0.85^b^	1.03^a^	1.10^a^	0.037	<0.001	<0.001	0.200	1.05^a^	1.05^a^	0.88^b^	0.167	<0.001	0.93	1.05	0.033	0.008
Milk protein (kg/d)	0.69^b^	0.91^a^	1.02^a^	0.043	<0.001	<0.001	0.275	0.88^b^	1.01^a^	0.73^c^	0.045	<0.001	0.81	0.94	0.041	0.011
Milk lactose (kg/d)	0.98^b^	1.20^a^	1.30^a^	0.056	<0.001	0.012	0.365	1.36^a^	1.12^b^	0.99^c^	0.054	<0.001	1.07	1.25	0.048	0.018
Milk fat (g/kg)	41.9	40.4	39.7	1.37	0.749	0.486	0.777	38.5^b^	40.8^a^	42.7^a^	1.34	0.022	41.3	40.0	1.13	0.792
Milk protein (g/kg)	34.0	35.2	35.9	1.09	0.440	0.213	0.795	31.5^c^	38.5^a^	35.1^b^	0.79	<0.001	35.1	34.9	0.89	0.886
Milk lactose (g/kg)	47.2	45.9	46.3	0.98	0.547	0.465	0.414	48.8^a^	43.0^a^	47.5^b^	0.71	<0.001	46.5	46.4	0.79	0.895
Milk energy (MJ/kg)	3.14	3.09	3.08	0.057	0.863	0.600	0.907	3.00^b^	3.12^a^	3.19^a^	0.055	0.002	3.11	3.10	0.047	0.968

Feed efficiency parameters																
Residual feed intake (kg/d)^6^	−1.18	−0.68	−1.13	0.232	0.298	0.910	0.125	−0.91	−1.22	−0.86	0.233	0.493	−0.85	−1.15	0.189	0.293
Milk/DMI	1.26	1.41	1.44	0.047	0.080	0.042	0.325	1.51^a^	1.42^b^	1.19^c^	0.042	<0.001	1.38	1.36	0.041	0.659
ECMY/DMI	1.27^b^	1.40^a^	1.42^a^	0.037	0.010	0.005	0.183	1.45^a^	1.41^a^	1.22^b^	0.032	<0.001	1.37	1.35	0.031	0.517
Milk solids/DMI^7^ (g/kg)	154^b^	170^a^	174^a^	4.6	0.020	0.009	0.269	178^a^	172^a^	149^b^	5.2	<0.001	168	164	3.9	0.367
Milk energy (E_L_)/DMI (MJ/DMI kg)	3.93^b^	4.33^a^	4.39^a^	0.100	0.010	0.005	0.183	4.49^a^	4.38^a^	3.77^b^	0.101	<0.001	4.25	4.18	0.097	0.517
Milk/concentrate DMI (kg/kg)	2.18	2.45	2.49	0.080	0.088	0.048	0.308	2.60^a^	2.45^b^	2.08^c^	0.072	<0.001	2.37	2.38	0.069	0.936
Milk fat/DMI (g/kg)	52.1	56.2	56.0	1.73	0.211	0.138	0.334	57.2^a^	56.9^a^	50.2^b^	1.64	0.006	56.1	53.5	1.420	0.203
Milk fat/concentrate DMI (g/kg)	90.5	97.3	96.6	2.98	0.209	0.249	0.301	98.7^a^	98.3^a^	87.5^b^	2.85	0.014	96.2	93.4	2.46	0.406
Milk protein/DMI (g/kg)	42.4^b^	49.2^a^	51.9^a^	1.99	0.011	0.004	0.391	47.3^b^	54.6^a^	41.7^c^	1.80	<0.001	48.4	47.3	1.81	0.660
Milk protein/concentrate DMI (g/kg)	73.7^b^	85.3^a^	89.1^a^	3.48	0.012	0.004	0.389	81.5^b^	94.4^a^	72.7^b^	3.16	<0.001	83.1	82.6	3.15	0.919
Milk/CP DMI (kg/kg)	10.3^a^	9.36^a^	8.04^b^	0.335	0.001	<0.001	0.657	10.2^a^	9.67^a^	7.88^b^	0.329	<0.001	9.30	9.19	0.315	0.698

Abbreviations: BCS = body condition score; DMI = DM intake; ECMY = energy corrected milk yield.

1 CP concentrations for LCP, MCP and HCP were 122, 151 and 181 g/kg DM, respectively.

2 Significances were declared at *P* < 0.05. Means within a row and fixed factor with different upper-case letters are significantly different according to Fisher’s protected least significant difference test (*P* < 0.05).

3 Early = 70–90 days; Mid = 150–170 days; Late = 230–250 days.

4 n is the number of records used to calculate means ± SE and *P*-values.

5 Significance for Linear (L) and Quadratic (Q) effects.

6 Residual feed intake = actual DMI (kg/d) – predicted DM requirement (kg/d).

7 Milk solids calculated as total fat, protein and lactose (g/d).

**Fig. 1:**
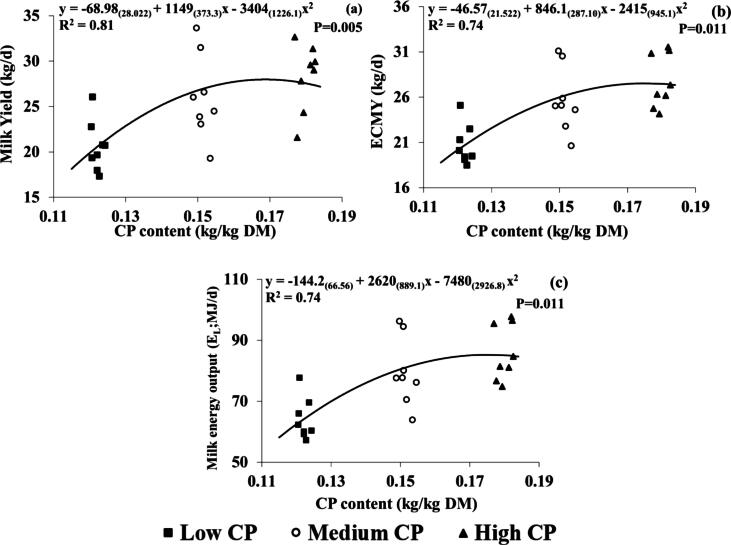
Quadratic relationship between dietary CP content (kg/kg DM) and (a) milk yield (kg/d) (b) Energy corrected milk yield (ECMY) (kg/d) and (c) Milk energy output (E_L_; MJ/d), for individual cows throughout lactation. Numbers in parentheses represent SEs. R^2^ is conditional on the presence of the random effects. Black squares, white circles and black triangles represent the low (122 g/kg DM), medium (151 g/kg DM) and high (181 g/kg DM) treatment groups, respectively.

When compared to the LCP group, milk, ECMY, fat, protein and lactose yields (kg/d) were higher for the MCP (+5.5, +5.0, +0.18, +0.22, and +0.22 kg/d) and HCP groups (+7.7, +7.1, +0.25, +0.33, and +0.32 kg/d) (Table 2) and were the only milk production and composition variables significantly (and linearly) affected by CP concentration. The correlations between dietary CP concentration (kg/kg DM) and productivity parameters are shown in Fig. 1. Regression analysis found significant quadratic relationships between milk yield (*P* = 0.005, R^2^; 0.81), ECMY (*P* = 0.011, R^2^; 0.74) and E_L_ (*P* = 0.011, R^2^; 0.74) and CP concentration (kg/kg DM).

**Fig. 2:**
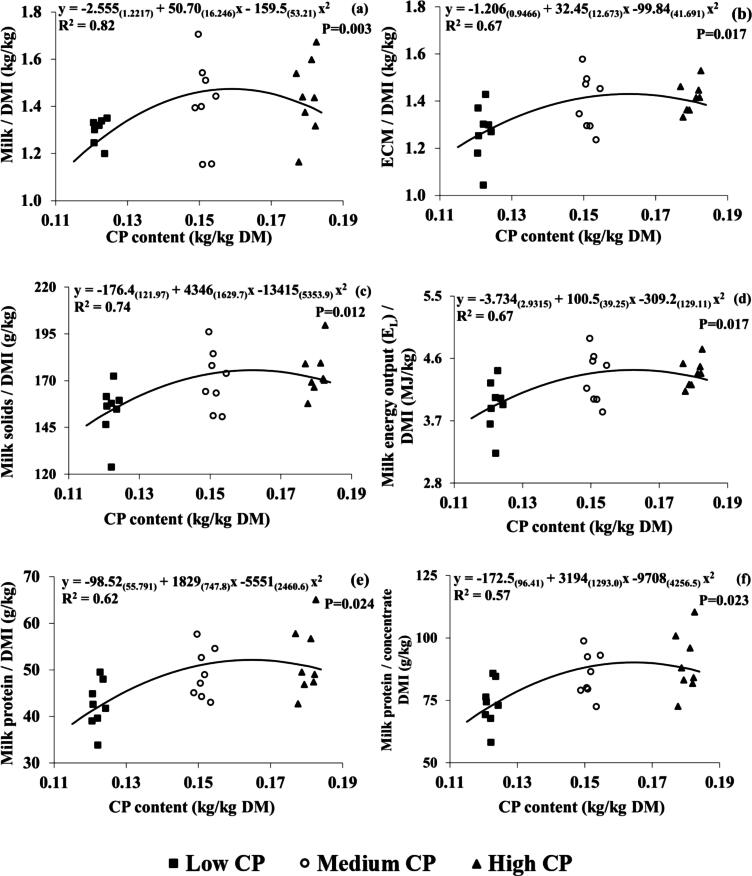
Relationship between dietary CP content (kg/kg DM) and (a) milk / DM intake (DMI) (kg/kg) (b) energy corrected milk (ECM) / DMI (kg/kg), (c) milk solids / DMI (g/kg), (d) milk energy output / DMI (MJ/kg), (e) milk protein / DMI (g/kg), (f) milk protein / concentrate DMI (g/kg) for individual cows throughout lactation. Numbers in parentheses represent SEs. R^2^ is conditional on the presence of the random effects. Black squares, white circles and black triangles represent the low (122 g/kg DM), medium (151 g/kg DM) and high (181 g/kg DM) treatment groups, respectively.

No differences were detected between CP concentration for milk/DMI (*P* = 0.080) or milk/concentrate DMI (*P* = 0.088); however, there were linear effects (*P* = 0.042 and *P* = 0.048) (Table 2). When compared with the LCP group, cows in the MCP and HCP groups showed higher efficiencies for ECMY (+0.13 and +0.15 kg/kg DMI), milk solids (+16 and +20 g/kg DMI), milk energy (+0.4 and 0.5 MJ/kg DMI), milk protein per kg DMI (+6.8 and 9.5 g/kg DMI), and milk protein per kg concentrate DMI (+12 and 15 g/kg) (Table 2). CP efficiency (kg milk/kg CP) was higher in LCP and MCP compared to the HCP group (+2.3 and +1.3 kg/kg) (Table 2). These differences were also linear. All other feed efficiency parameters were not significantly affected by dietary CP concentrations (Table 2). Correlations between dietary CP concentration (kg/kg DM) and feed efficiency parameters are shown in Fig. 2. Regression analysis found significant quadratic relationships between milk/DMI (*P* = 0.003, R^2^; 0.82), ECMY/DMI (*P* = 0.017, R^2^; 0.67), milk solids/DMI (0.012, R^2^; 0.74), E_L_/DMI (*P* = 0.017, R^2^; 0.67), milk protein/DMI (*P* = 0.024, R^2^; 0.62), milk protein/concentrate DMI (*P* = 0.023, R^2^; 0.57) and CP concentration (kg/kg DM).

#### Effect of stage of lactation

Animal LW varied between the stages of lactation, increasing by +27 kg from early to mid and by +49 kg from mid to late (Table 2). Animal body condition score was highest in late, compared to mid (+0.18) and early (+0.27) lactation. All feed intake parameters significantly differed between stages of lactation. Total DMI was highest in early and mid−lactation (+0.80 kg/d) compared to late−lactation. Grass silage DMI was higher in mid−lactation compared to late−lactation (+0.33 kg/d) (Table 2). Maize silage DMI was higher in early−lactation compared to mid−lactation (+0.29 kg/d). Concentrate DMI was higher in early and mid−lactation compared to late−lactation (+0.60 kg/d) (Table 2). Stage of lactation also significantly affected all milk production, milk composition and efficiency parameters (Table 2), except for residual feed intake. When compared with late−lactation, early and mid−lactation showed higher milk yield (+7.0 and +5.3 kg/d, respectively), ECMY (+5.9 and +5.2 kg/d, respectively) and fat yield (+0.17 for both early and mid−lactation) (Table 2). Milk protein yield (kg/d) increased from early to mid (+0.13) and was lowest for late compared to both early (−0.17) and mid−lactation (−0.28). Lactose yield (kg/d) decreased from early to mid−lactation (−0.24) and further decreased from mid− to late−lactation (−0.13). Milk fat content was lower in early−lactation than in mid− and late−lactation (−2.3 and −4.2 g/kg, respectively) (Table 2). Milk protein content was highest in mid−lactation, lowest in early−lactation and showed intermediate values in late−lactation (+3.4 and +3.6 g/kg from late to mid and early to late, respectively) (Table 2). Milk lactose concentrations were highest in early and late−lactation (+5.8 and +4.5 g/kg) compared to mid−lactation (Table 2). Milk GE (MJ/kg) was highest in mid and late compared to early (+0.12 and +0.19 MJ/kg) lactation. Milk (kg/kg DMI) and feed concentrate efficiency (kg milk/kg concentrate DMI) were higher in early compared to mid−lactation (+0.09 and +0.15 kg/kg respectively), and higher during mid− compared to late−lactation (+0.23 and 0.37 kg/kg respectively) (Table 2). During early and mid−lactation, ECMY/DMI were respectively 0.23 and 0.19 kg/kg higher when compared to late−lactation The corresponding data for milk solids/DMI were respectively (+29 and +23 g/kg), milk energy/DMI (+0.72 and +0.61 MJ/kg), fat/DMI (+7.0 and 6.7 g/kg), and fat/concentrate DMI (+11.2 and 10.8 g/kg) (Table 2). Milk protein efficiency (milk protein/DMI; g/kg) was highest in mid−lactation compared to early (+7.30 g /kg DMI) and lower in late compared to early−lactation (−5.6 g/kg DMI) (Table 2). Protein concentrate efficiency (milk protein/concentrate DMI; g/kg) was higher in mid−lactation compared with both early and late−lactation (+12.9 and +21.7 g/kg DMI). During early and mid−lactation, CP efficiency (Milk/CP intake) was higher compared to late−lactation (+2.32 and +1.79 kg/kg CP) (Table 2).

#### Effect of parity

All feed intake and LW measures significantly differed between parity, except for DM digestibility (Table 2). Multiparous cows were heavier (+84 kg) and had higher body condition score (+0.16) than primiparous cows. Multiparous had higher total DMI (+2.9 kg/d) and higher intakes of grass silage, maize silage and concentrate (+0.89, +0.52 and +1.49 kg/d) than primiparous cows. No differences between parity were found for milk production and composition measures except for milk yield and ECMY, which were higher for multiparous cows compared to primiparous cows (+3.8 and +3.7 kg/d, respectively).

#### Effect of CP concentration × stage of lactation

Analysis for pairwise comparisons for interactions between CP concentration × stage of lactation showed no interactions (Supplementary Table S1). However, linear interactions were found for total DMI (*P* = 0.041), concentrate DMI (*P* = 0.048), CP intake (*P* = 0.037), milk yield (*P* = 0.020), and ECMY (*P* = 0.040). Numerically, cows in the HCP during early−lactation had the highest total DMI (20.5 kg/d) and cows in the LCP had the lowest intakes during early−lactation (16.2 kg/d). Concentrate DMI and CP intake was highest for cows in LCP during late−lactation (12.0 and 3.69 kg/d) and was lowest for cows in the LCP treatment during early−lactation (9.36 and 1.96 kg/d). Milk yield and ECMY were highest in early−lactation for the HCP treatment (32.9 and 31.1 kg/d) and lowest in late−lactation for the LCP treatment (18.2 and 18.7 kg/d). Data reported in Supplementary Table S1.

#### Effect of CP concentration × parity

**Fig. 3:**
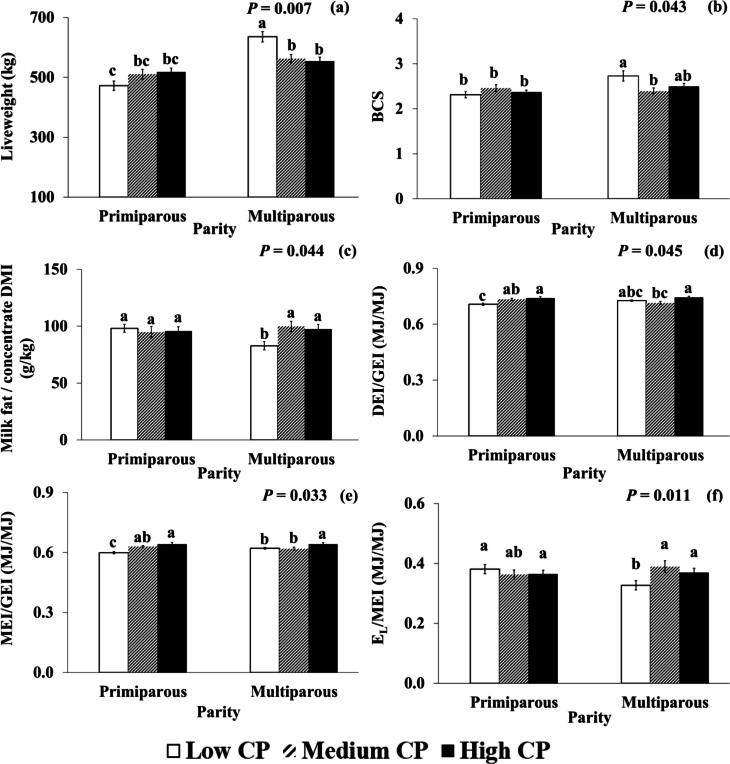
Interactions of CP level × parity for (a) Liveweight (kg) (b) body condition score (BCS) (c) Milk fat / concentrate DM intake (DMI) (g/kg) (d) Digestible energy intake (DEI)/Gross energy intake (GEI) (MJ/MJ) (e) metabolisable energy intake (MEI)/GEI (MJ/MJ) (f) milk energy output (E_L_)/MEI (MJ/MJ) for primiparous and multiparous cows throughout lactation offered three experimental diets containing differing CP levels (high: 181 (g/kg DM), black bar; medium: 151 (g/kg DM), patterned bar; low: 122 (g/kg DM), white bar). Bars with different upper-case letters are significantly different according to Fisher’s protected least significant difference test (*P* < 0.05).

Significant and linear CP concentration × parity interactions were found for LW. Significant effects were also found for body condition score, DM digestibility and fat concentrate efficiency. Linear effects were found for fat efficiency, and quadratic effects were found for body condition score and DM digestibility (Supplementary Table S1). Multiparous cows in the LCP group had higher LW than multiparous cows in HCP and MCP treatments (+82 and +73 kg, respectively) but a similar difference was not observed for primiparous cows (Fig. 3). Multiparous cows in the LCP treatment had higher body condition score compared to multiparous cows in the MCP treatments (+0.33), but not in the HCP treatment, but there were no differences observed for primiparous cows. DM digestibility was lower in LCP treatment (−2.6% and −3.1%) compared to MCP and HCP treatment, but there was no difference for multiparous cows. Multiparous cows in the LCP treatment had significantly lower fat concentrate efficiency than multiparous cows in the HCP and MCP treatments (−15 and −17 g fat/kg concentrate DMI), while fat concentrate efficiency in primiparous cows was not significantly affected by lactation stage. Numerically, fat efficiency was highest in early−lactation for the MCP treatment (59.1 g/kg) and lowest in late−lactation for the LCP treatment (47.6 kg/d).

#### Effect of stage of lactation × parity

**Fig. 4:**
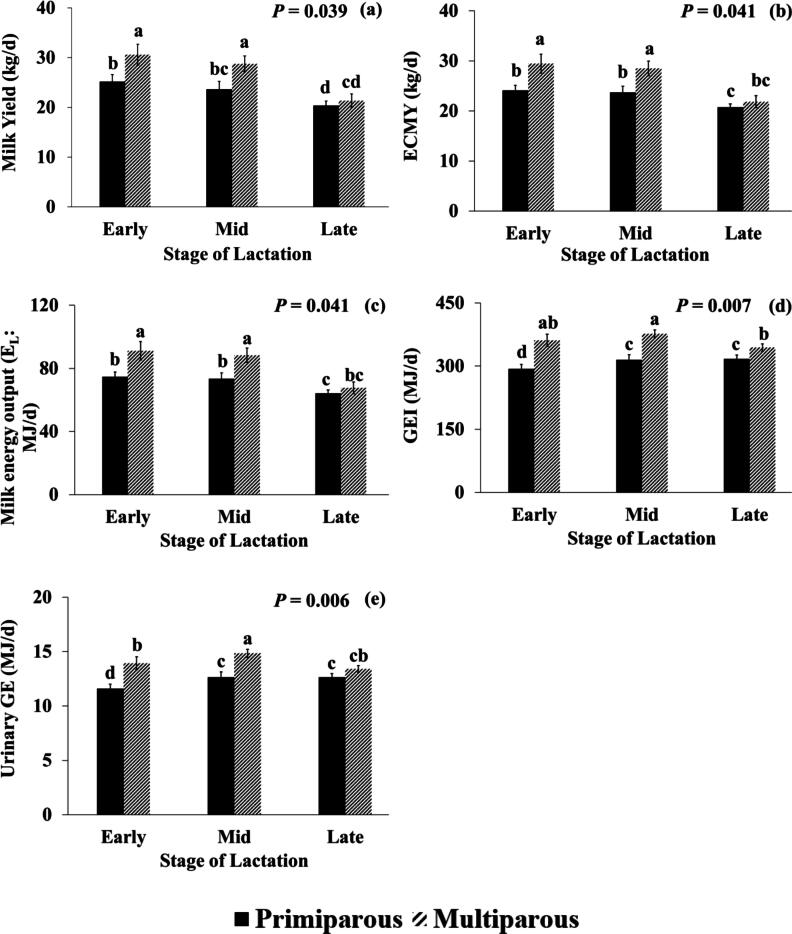
Interactions of stage of lactation × parity for (a) Milk yield (kg/d) (b) Energy corrected milk yield (ECMY) (kg/d) (c) Milk energy output (E_L_) (MJ/d) (d) Gross energy intake (GEI) (MJ/d) (e) Urinary gross energy (GE) (MJ/d) for primiparous (black bar) and multiparous cows (patterned bar) throughout lactation (early, mid and late). Bars with different upper-case letters are significantly different according to Fisher’s protected least significant difference test (*P* < 0.05).

Significant stages of lactation × parity interactions were found for all feed intake measures except for, grass silage DMI and CP intake (Supplementary Table S1). Additionally, significant effects were also found for total DMI, concentrate DMI, CP intake, DM digestibility and ECMY. Multiparous cows in early and mid-lactation had higher milk yields (+9.2 and +7.4 kg/d) and ECMY (+8.1 and +6.7 kg/d) than primiparous cows in early (+3.6 kg/d for milk yield and +4.4 kg/d for ECMY, respectively) and mid− (+5.1 kg/d for milk yield and +4.8 kg/d for ECMY, respectively) lactation but there was no significant difference between multiparous and primiparous cows in late−lactation. Both multiparous and primiparous cows in late−lactation had lower milk yield and ECMY compared to early (−9.2 kg/d and 7.6 kg/d for multiparous and −4.8 kg/d and 3.3 kg/d for primiparous cows) and mid−lactation (−7.4 kg/d and 6.7 kg/d for multiparous and −3.2 kg/d and 2.9 kg/d for primiparous cows) (Fig. 4).

### Energy use efficiency and methane production

#### Effect of CP concentration

**Fig. 5:**
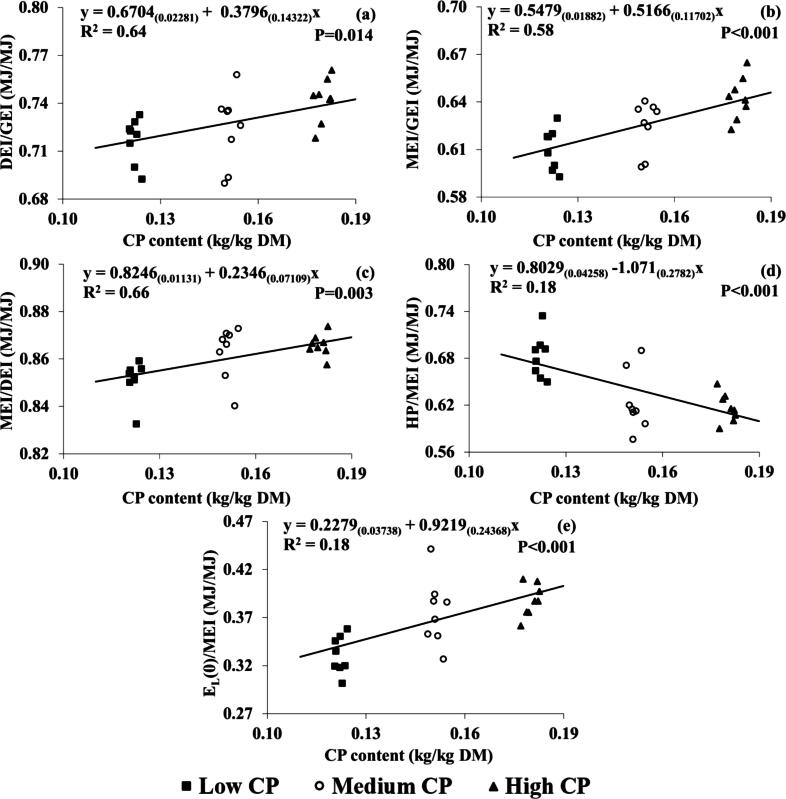
Relationship between dietary CP content (kg/kg DM) and (a) Digestible energy intake (DEI) / gross energy intake (GEI) (MJ/MJ), (b) metabolisable energy intake (MEI) / gross energy intake (GEI) (MJ/MJ), (c) MEI/DEI (MJ/MJ), (d) heat production (HP) / MEI intake (MJ/MJ), (e) milk energy output, corrected for 0 energy balance (E_L(0)_/ MEI (MJ/MJ) for individual cows throughout lactation. Numbers in parentheses represent SEs. R2 is conditional on the presence of the random effects. Black squares, white circles and black triangles represent the low (122 g/kg DM), medium (151 g/kg DM) and high (181 g/kg DM) treatment groups, respectively.

There were significant and linear effects of dietary CP concentration on GEI, DEI, MEI, faecal GE, urinary GE, HP and milk energy (Table 3). Compared with the LCP group, MCP and HCP groups had higher GEI (+39 and +62 MJ/d, respectively), faecal GE (+9.2 and +8.7 MJ/d, respectively), urinary GE (+1.5 and 2.6 MJ/d, respectively) and milk energy output (+15.5 and +22 MJ/d) (Table 3). The HCP group had a significantly higher HP than the LCP group (+18 MJ/d), but the differences in HP between HCP and MCP groups or between MCP and LCP groups did not differ significantly. Digestible energy intake and MEI were higher in the HCP compared to MCP (+24 and +22 MJ/d), and MCP was higher than the LCP (+30 and +28 MJ/d) (Table 3). Results for pairwise comparison showed no significant differences between means for RE (*P* = 0.096), but showed significant linear differences (*P* = 0.033). The effect of dietary CP concentration was significant and linear for all energy use efficiency parameters, except for E_L_/MEI (Table 3) and K_L_. The HCP group had a higher DEI/GEI compared to the LCP and MCP groups (+0.02 MJ/MJ). The MEI/GEI increased as CP concentration increased (+0.01 from LCP to MCP and +0.02 from MCP to HCP) (Table 3). The MCP and HCP treatments resulted in higher MEI/DEI (+0.01 and +0.02 MJ/MJ, respectively) and E_I(0)/_MEI (+0.05 and +0.06 MJ/MJ, respectively) compared to the LCP treatment (Table 3). The LCP treatment group had higher HP/MEI (+0.06 MJ/MJ) compared to the MCP and HCP groups (Table 3). Regression analysis found positive linear relationships between DEI/GEI (*P* = 0.014, R^2^; 0.64), MEI/GEI (*P* < 0.001, R^2^; 0.58), MEI/DEI (*P* = 0.003, R^2^; 0.66), E_L(0)/_MEI (*P* < 0.001, R^2^; 0.18) and CP concentration (kg/kg DM) and a negative linear relationship for HP/MEI (*P* < 0.001, R^2^; 0.18). These correlations between dietary CP concentration (kg/kg DM) and energy use efficiency parameters are shown in Fig. 5.

**Table 3:** Means ± SE and *P*-values effect of dietary CP concentration (low/medium/high), stage of lactation, and their interactions on energy intake and outputs, energy use efficiency and methane production parameters in lactating dairy cows.

	CP concentration (CP)							Stage of Lactation (SL)					Parity			
	LCP^1^	MCP^1^	HCP^1^	SE	*P*-value^2^	*P*-value^2^		Early^3^	Mid^3^	Late^3^	SE	*P*-value^2^	Primiparous	Multiparous	SE	*P*-value^2^
Item	n = 24^4^	n = 24^4^	n = 24^4^			L^5^	Q^5^	n = 24^4^	n = 24^4^	n = 24^4^			n = 36^4^	n = 36^4^		
Energy parameters (MJ/d)																
GEI	301^b^	340^a^	363^a^	7.9	<0.001	<0.001	0.330	327^b^	346^a^	331^b^	9.2	0.004	309	361	6.4	<0.001
DEI	216^c^	246^b^	270^a^	6.1	<0.001	<0.001	0.587	234^b^	255^a^	242^b^	7.2	<0.001	225	263	5.3	<0.001
MEI	184^c^	212^b^	234^a^	5.4	<0.001	<0.001	0.475	202^b^	221^a^	207^b^	6.6	<0.001	193	227	4.8	<0.001
Faecal GE	84.8^b^	94.0^a^	93.5^a^	2.57	0.034	0.022	0.180	93.0	90.9	88.5	2.68	0.285	83.8	97.8	1.855	<0.001
Urinary GE	11.8^b^	13.3^a^	14.4^a^	0.29	<0.001	<0.001	0.475	12.8^b^	13.7^a^	13.0^b^	0.35	<0.001	12.3	14.1	0.26	<0.001
CH_4_-E	20.1	20.3	21.7	0.67	0.335	0.233	0.383	19.3^c^	20.7^b^	22.1^a^	0.64	<0.001	19.3	22.1	0.50	0.007
HP^6^	125^b^	132^ab^	143^a^	3.1	<0.001	<0.001	0.525	128^b^	138^a^	133^a^	3.4	0.001	123	144	2.2	<0.001
E_L_	64.1^b^	79.6^a^	86.1^a^	2.80	<0.001	<0.001	0.107	82.9^a^	80.9^a^	66.0^b^	3.00	<0.001	70.8	82.4	2.54	<0.001
Retained energy	−4.69	0.72	4.26	3.307	0.096	0.033	0.767	−9.08^c^	1.55^b^	7.82^a^	3.070	0.002	−0.30	0.49	2.745	0.813

Energy use efficiency																
DEI/GEI	0.72^b^	0.72^b^	0.74^a^	0.005	0.013	0.004	0.405	0.71^b^	0.74^a^	0.73^a^	0.005	0.001	0.73	0.73	0.004	0.925
MEI/GEI	0.61^c^	0.62^b^	0.64^a^	0.004	<0.001	<0.001	0.750	0.62^b^	0.64^a^	0.63^ab^	0.005	0.002	0.62	0.63	0.004	0.518
MEI/DEI	0.85^b^	0.86^a^	0.87^a^	0.002	0.001	<0.001	0.143	0.86^a^	0.86^a^	0.85^b^	0.003	0.004	0.86	0.86	0.002	0.274
HP/MEI	0.68^a^	0.62^b^	0.62^b^	0.011	<0.001	<0.001	0.075	0.64	0.63	0.65	0.013	0.695	0.64	0.64	0.010	0.877
E_L_/MEI	0.35	0.38	0.37	0.011	0.221	0.313	0.155	0.41^a^	0.37^b^	0.32^c^	0.009	<0.001	0.37	0.36	0.009	0.474
E_L(0)/_MEI	0.33^b^	0.38^a^	0.39^a^	0.010	<0.001	<0.001	0.061	0.37	0.37	0.36	0.011	0.515	0.37	0.37	0.009	0.918
K_L_	0.56	0.58	0.56	0.001	0.308	0.651	0.145	0.57	0.56	0.57	0.012	0.714	0.57	0.56	0.010	0.389

Methane parameters																
CH_4_ (g/d)	363	367	391	12.0	0.362	0.250	0.401	348^c^	374^b^	399^a^	11.5	<0.001	349	399	9.0	0.008
CH_4_/DMI (g/kg)	22.2	20.1	20.1	0.66	0.068	0.046	0.216	19.1^c^	20.5^b^	22.9^a^	0.62	<0.001	21.1	20.5	0.57	0.511
CH_4_/DDMI (g/kg)	30.7^a^	27.5^b^	26.9^b^	0.88	0.018	0.009	0.218	26.2^b^	27.7^b^	31.2^a^	0.83	<0.001	28.8	28.0	0.76	0.479
CH_4_/Milk yield (g/kg)	18.3	14.9	14.6	1.00	0.051	0.032	0.222	13.0^c^	14.9^b^	19.9^a^	0.85	<0.001	15.9	16.0	0.85	0.815
CH_4_/ECMY (g/kg)	17.9^a^	14.8^b^	14.5^b^	0.81	0.011	0.008	0.120	13.4^b^	14.6^b^	19.2^a^	0.68	<0.001	15.7	15.9	0.70	0.769
CH_4_-E /GEI (MJ/MJ)	0.067^a^	0.060^b^	0.060^b^	0.0018	0.030	0.020	0.174	0.060^b^	0.060^b^	0.067^a^	0.0019	0.002	0.063	0.062	0.0016	0.562
CH_4_-E /DEI (MJ/MJ)	0.094^a^	0.083^b^	0.080^b^	0.002	0.004	0.002	0.158	0.084^b^	0.082^b^	0.092^a^	0.0025	0.003	0.087	0.085	0.0022	0.487
CH_4_-E /MEI (MJ/MJ)	0.110^a^	0.096^b^	0.093^b^	0.003	0.004	0.002	0.161	0.097^b^	0.095^b^	0.107^a^	0.0031	0.004	0.101	0.098	0.0027	0.458

Abbreviations: GEI = gross energy intake, GE = gross energy, HP = heat production, DEI = digestible energy intake, MEI = metabolisable energy intake, E_L_ = milk energy output (MJ/d), E_L(0)_ = E_L_ adjusted to 0 energy balance, K_L_ = efficiency of utilisation of ME for milk production; DMI = DM intake; DDMI = digestible DMI, ECMY = energy corrected milk yield, CH_4_-E = CH_4_ energy.

1 CP concentrations for LCP, MCP and HCP were 122, 151 and 181 g/kg DM, respectively.

2 Significances were declared at *P* < 0.05. Means within a row and fixed factor with different upper-case letters are significantly different according to Fisher’s protected least significant difference test (*P* < 0.05).

3 Early = 70–90 days; Mid = 150–170 days; Late = 230–250 days.

4 n is the number of records used to calculate means ± SE and *P*-values.

5 Significance for Linear (L) and Quadratic (Q) effects.

6 Heat production was calculated based on O_2_ consumption, CO_2_ and CH_4_ production and urinary N excretion (**UN**) using the equation of Brouwer (1965); [(16.18 × O_2_) + (5.16 × CO_2_) − (2.42 × CH_4_) − (5.9 × UN)]/1 000.

**Fig. 6:**
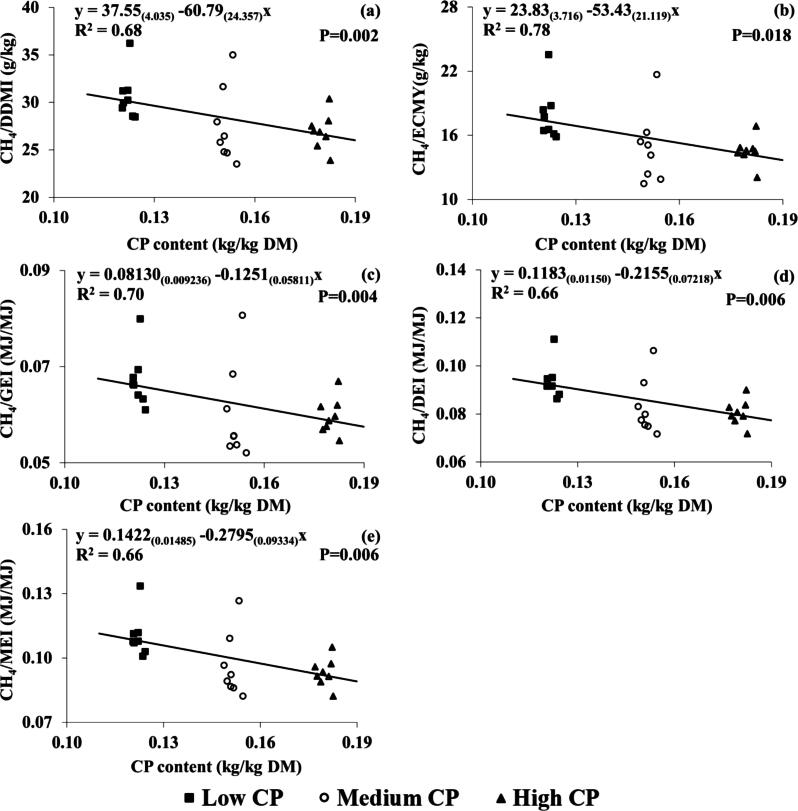
Relationship between dietary CP content (kg/kg DM) and (a) CH_4_/ digestible DM intake (DDMI) (g/kg), (b) CH_4_/energy corrected milk yield (ECMY) (g/kg), (c) CH_4_ energy/ gross energy intake (GEI) (MJ/MJ), (d) CH_4_ energy/ digestible energy intake (DEI) (MJ/MJ) and (e) CH_4_ energy output (MJ) / metabolisable energy intake (MEI) (MJ) for individual cows throughout lactation. Numbers in parentheses represent SEs. R^2^ is conditional on the presence of the random effects. Black squares, white circles and black triangles represent the low (122 g/kg DM), medium (151 g/kg DM) and high (181 g/kg DM) treatment groups, respectively.

Pairwise comparison found significant effects of dietary CP concentration on all CH_4_ production parameters, except for CH_4_ (g/d), CH_4_/DMI (g/kg) and CH_4_/milk (g/kg). Significant linear effects of dietary CP concentration were also found on all CH_4_ production parameters, except for CH_4_ (g/d) (Table 3). The LCP group showed higher CH_4_ output than MCP and HCP per kg digestible DMI (+3.2 and +3.8 g/kg), per kg ECMY (+3.1 and +3.4 g/kg), per GEI (+0.007 MJ/MJ), per DEI (+0.011 and 0.014 MJ/MJ) and per MEI (+0.014 and +0.017 MJ/MJ). Regression analysis found negative linear relationships between CH_4_/digestible DMI (*P* = 0.002, R^2^; 0.68), CH_4_/ECMY (*P* = 0.018, R^2^; 0.78), CH_4_/GEI (*P* = 0.004, R^2^; 0.70), CH_4_/DEI (*P* = 0.006, R^2^; 0.66), CH_4_/MEI (*P* = 0.006, R^2^; 0.66) and CP concentration (kg/kg DM). These correlations between dietary CP concentration (kg/kg DM) and CH_4_ production parameters are shown in Fig. 6.

#### Effect of stage of lactation

Stage of lactation significantly impacted all energy parameters, except for faecal GE (Table 3). Gross energy intake, DEI, MEI, urinary GE and HP were higher in mid−lactation than in early (+19, +21, +19, +0.9 and +10 MJ/d, respectively) and late−lactation (+15, +13, +14, +0.7 and +5 MJ/d, respectively) (Table 3). Methane energy and RE were higher with increasing stages of lactation (+1.4 and +10.6 MJ/d from early to mid and +1.4 and +6.3 MJ/d for CH_4_-E and RE, respectively). Milk energy output was lower in late−lactation than in early and mid−lactation (−16.9 and −14.9 MJ/d, respectively) (Table 3). All energy use efficiency parameters were significantly impacted by the stage of lactation, except for HP/MEI and E_L(0)_/MEI (Table 3). During mid−lactation, DEI/GEI and MEI/GEI were higher than in early−lactation (+0.03 and +0.02 MJ/MJ, respectively) (Table 3) but MEI/DEI was higher in early and mid, compared to late−lactation (+0.01 MJ/MJ) (Table 3). The E_L_/MEI decreased throughout lactation by −0.04 MJ/MJ from early to mid and by −0.05 MJ/MJ from mid− to late−lactation (Table 3). All CH_4_ parameters showed significant variation across lactation (Table 3). Overall CH_4_ output increased across lactation by +26 g/d from early to mid and by +25 g/d from mid− to late−lactation, along with CH_4_ g per kg DMI (+1.4 from early to mid and +2.4 g/kg from mid− to late−lactation) and per kg milk yield (+1.9 from early to mid and +5.0 g/kg from mid to late, respectively). During late−lactation CH_4_ output was higher than both early and mid−lactation per kg digestible DMI (+5.0 and +3.5 g/kg, respectively), ECMY (+5.8 and +4.6 g/kg, respectively), GEI (+0.007 MJ/MJ), DEI (+0.008 and 0.010 MJ/MJ), and MEI (+0.010 and 0.012 MJ/MJ).

#### Effect of parity

Significant effects for parity were found for all energy parameters (Table 3). Multiparous cows had higher (MJ/d) GEI (+52), DEI (+38), MEI (+34), faecal energy (+14), urinary energy (+1.8), CH_4_ energy (+2.8), heat production (+21), and milk energy (+11.6) when compared with primiparous cows. However, no significant differences were found for retained energy (Table 3). There were no statistically significant differences for energy use efficiency or CH_4_ production parameters, except for CH_4_ g/d, with multiparous cows producing 50 g/d more total CH_4_ than primiparous cows (Table 3).

#### Effect of CP concentration × stage of lactation

There were no CP concentration × stage of lactation interactions for energy use efficiency parameters or CH_4_ production parameters (Supplementary Table S2). However, the analysis showed linear interactions for GEI (*P* = 0.033), DEI (*P* = 0.018), MEI (*P* = 0.022), urinary GE output (*P* = 0.022) and E_L_ (*P* = 0.040). Numerically, GEI, DEI, MEI and urinary GE output was highest for the HCP treatment during mid−lactation (374, 284, 247 and 14.5 MJ/d) and lowest for the LCP treatment during early−lactation (283, 200, 170 and 11.0 MJ/d). Numerically, E_L_ was highest for the HCP treatment during early −lactation (96.2 MJ/d) and lowest for the LCP treatment during late−lactation (57.9 MJ/d).

#### Effect of CP concentration × parity

Significant effects of CP concentration × parity interactions were found only for some energy use efficiency parameters; DEI/GEI, MEI/GEI and E_L_/MEI (Supplementary Table S2; Fig. 3). Primiparous cows had higher DEI/GEI (+0.03 MJ/MJ) in HCP than in LCP while multiparous cows had higher DEI/GEI (+0.03 MJ/MJ) in HCP than in MCP. Both multiparous and primiparous cows in the HCP treatment had higher MEI/GEI than in MCP (+0.02 for multiparous and +0.01 for primiparous cows; MJ/MJ) and LCP treatments (+0.02 for multiparous and +0.04 for primiparous cows; MJ/MJ), although the difference between HCP with LCP and MCP was not significant in primiparous cows. These interactions were also quadratic for DEI/GEI and MEI/GEI. Multiparous cows showed a lower E_L_/MEI in the LCP than in MCP and HCP (−0.06 vs MCP and – 0.04 vs HCP) but similar differences were not observed for primiparous cows (Fig. 3). Interactions for E_L_/MEI were also linear and quadratic.

#### Effect of stage of lactation × parity

The interaction effect of stage of lactation × parity was significant for GEI, DEI, MEI, urinary energy, and E_L_ but did not significantly affect any other energy and energy use efficiency parameters (Supplementary Table S2; Fig. 4). Multiparous cows had higher GEI than primiparous in early (+67.6 MJ/d), mid− (+61.9 MJ/d) and late− (+26.5 MJ/d) lactation with their relative difference becoming lower in late−lactation. The same was observed for DEI with multiparous having higher DEI than primiparous cows in early (+49.0 MJ/d) mid (+47.0 MJ/d) and late−lactation (+18.0 MJ/d). Multiparous cows had higher MEI than primiparous cows in early (+44.0 MJ/d) and mid (+42.0 MJ/d) but MEI was similar between parity in late−lactation. Urinary GE was higher for multiparous than primiparous cows in early (+2.3 MJ/d) and mid (+2.2 MJ/d) with urinary GE being similar between parity in late−lactation. Multiparous cows in early and mid−lactation produced higher energy yield than primiparous cows in early (+17 and +14 MJ/d), mid− (+18 and +15 MJ/d) and late−lactation (+27 and +24 MJ/d), but there was no significant difference in late−lactation (Fig. 4).

## Discussion

### Effect of CP concentration

#### Feed intake, productivity, and feed efficiency

Previous investigations have observed depressed DMI as CP concentration was reduced (Broderick, 2003, Broderick et al., 2009, Barros et al., 2017, Letelier et al., 2022), in agreement with the findings of the current study. Observed increases in DMI are primarily in response to rumen degradable protein supply and subsequent increased DM digestibility resulting in faster transit time and reduction in rumen distention (Roffler et al., 1986, Allen, 2000). Interestingly, DMI in the current study were similar for MCP (15.1% CP in DM) and HCP (18.1% CP in DM) groups. Similar results were found by Hynes et al., 2016 in which DMI was not affected by incremental increases in CP concentrations of 16.9% DM, 17.6% DM and 18.3% DM. Previous investigations have suggested that the increasing rate in DM digestibility with increasing dietary CP concentration decreased after CP concentration reached up to a certain concentration (Roffler et al., 1986, Allen, 2000). This indicates that DMI may not differ significantly at different high concentrations of dietary CP. Thresholds, at which the rate of increase in DMI plateaus differs between studies and ranges between 15.1 and 18.3% CP (DM basis), were observed in the current study and previous investigations (Cabrita et al., 2007, Law et al., 2009, Hynes et al., 2016). Based on the results of the present and previous works, the variation in CP concentration around the range which is often fed to high−producing cows (16–18% CP; Webster, 2020) is unlikely to result in large differences in DMI; but can be reduced when diet CP concentrations drop any further (<15.1% CP DM) as seen in the current study. Additionally, other studies have observed interactions between CP concentration and stage of lactation; Letelier et al. (2022) found that DMI response to dietary CP concentration depended on stage of lactation. Early, mid-early, and late−lactation cows (86, 119, and 239 days in milk, respectively) had the greatest DMI when fed diets with 16.7% CP (32.9, 31.3, and 29.1 kg/d, respectively), whereas mid-late lactation cows (167 d) had the greatest DMI when fed a diet with 18.3% CP. The current study did find linear CP concentration × stage of lactation interactions for total DMI, but pairwise comparisons were not performed; numerically, cows in the HCP (18.1% CP DM) had the greatest DMI (20.5 kg/d) during early−lactation (70–90 days in milk) whilst cows in the MCP and LCP treatments had the greatest DMI (18.7 and 16.8 kg/d) during mid−lactation (150–170 kg/d).

The findings of higher milk yield and ECMY in MCP and HCP treatments in the current study are in line with previous investigations (Cabrita et al., 2007, Barros et al., 2017). Increased productivity in response to increasing dietary CP concentration has been primarily attributed to increased DMI, supported by the findings of higher DMI in MCP and HCP compared to the LCP treatments in the current study. As expected, the observed similarities in DMI between the MCP and HCP groups resulted in no further increases in milk and ECMY. This aligns with Broderick (2003) who observed no difference in milk yield between treatments of 16.7 and 18.4% CP concentration, although reducing dietary CP from 16.7 to 15.1% significantly reduced milk yield by 1.1 kg/d. Results from the current study suggest that further increases in CP exceeding 15.1% may not result in any beneficial increase in productivity. However, across lactation, the HCP diet resulted in animals producing 8 479 kg of milk compared to 7 839 kg and 6 314 kg for the MCP and LCP treatments over 305 days of lactation. Wu and Satter (2000) found no differences in overall milk yield (308 d lactation) between diets containing 17.4–17.9% and 19.3–17.9% CP (DM basis) (11 095 and 11 132 kg, respectively), but yield was 776 kg higher when animals were offered diets containing 17.4–16.0% CP (DM basis) (10 832 kg) compared to diets containing 15.4–16.0% (DM basis) (10 056 kg). These results suggest that reductions in CP below 16% may result in negative impacts on milk production across lactation, but further increases above 17.4% may not result in any beneficial increase in milk production. Results from regression analysis (Fig. 1a) suggest that there may be an undetected optimum CP concentration for milk yield between 15.1% and 18.1% CP DM. Milk yield and ECMY peaked (28.0 and 27.5 kg/d, respectively) at CP concentrations of 17.0% and 18.0%, respectively. Additionally, linear interactions of CP concentration × stage of lactation were found for DMI, milk and ECMY suggesting that responses to CP concentration could be dependent on days in milk. Differences between CP concentration for DMI, milk yield and ECMY were numerically higher in early–lactation compared to mid and late, suggesting that response to CP diminishes as lactation progresses, also suggested by Letelier et al. (2022). Law et al. (2009) similarly reported no detrimental effect on milk yield when CP was reduced after day 151 of lactation from 17.3 to 14.4% CP concentration. Thus, based on findings by Letelier et al. (2022) and Law et al. (2009), it is possible that a lower CP (between 15 and 17%) concentration could be offered particularly during later lactation without detrimental effects on productivity.

Previous work has observed higher DMI without a simultaneous increase in milk yield in response to increasing CP; Broderick (2003) reported no further increase in milk at 18.4% CP of DM compared to 16.7 and 15.1% but DMI at 18.4% was the highest of the three treatments resulting in lower feed efficiency (milk/DMI). Consequently, animals offered diets with 15.1 and 16.7% CP of DM produced 50 g and 40 g more milk per kg of DMI than animals offered diets with 18.4% CP of DM. The current study did not observe any impact of CP concentration on feed efficiency (milk/DMI) but this could have been due to the relatively low CP concentrations investigated compared to other investigations that have found differences in feed efficiency in response to differing CP concentration; i.e. 12.2, 15.1 and 18.1% CP (DM basis) in the current study vs 15.1, 16.7 and 18.4% CP (DM basis) by Broderick (2003). The results of Cabrita et al. (2007) also support the findings in the current study observing no difference in feed efficiency calculated as milk/DMI, ECMY/DMI, milk fat/DMI and milk protein/CP intake between diets containing 14.0 and 16.0% CP of DM. Considering that dairy cow diets typically contain between 16.0 and 18.0% CP (Webster, 2020), the considerable cost of protein and the poor N use efficiency (between 25 and 31% (Yang et al., 2022)), feeding practices at 18.0% CP of DM may pose an unnecessary risk for efficiency and profitability in dairy farms. Whilst a linear effect of CP concentration on milk/DMI was found, regression analysis suggested quadratic effects; milk/DMI increases until CP concentration reaches beyond 16% DM, after which milk/DMI was not improved and in fact had a negative impact. Regarding the potential lower end of dietary CP concentration, findings that MCP and HCP groups were more efficient converters of DMI to ECMY, milk solids (fat, protein and lactose) and milk energy are in agreement with Barros et al. (2017) observing higher feed efficiency (fat and protein corrected milk/DMI and ECMY/DMI) under diets containing 16.2 and 14.4% CP compared to 13.1 and 11.8%, with the 16.2 and 14.4% CP (DM basis) diets yielding similar results. The current study also found that MCP and HCP groups were more efficient converters of overall DMI to milk solids and milk energy and DMI from concentrates to milk protein, primarily attributed to the higher milk yields observed by these groups. Olmos Colmenero and Broderick (2006) suggested that feeding less than 16.0% CP of DM may not provide sufficient metabolisable protein for optimal milk production, but diets containing 17.0% CP of DM would not improve milk production further. Results from restricted maximum likelihood analysis in the current study, along with Barros et al. (2017), suggest that the optimal concentration for CP inclusion may be much lower (between 15.0 and 18.0% CP of DM as seen in the current study), considering the similarities in milk yield, ECMY and efficiency parameters (ECMY/DMI, milk solids/DMI and milk energy/DMI) between MCP and HCP treatments. However, cows used in Barros et al. (2017) were all in later lactation (150 days or greater). Thus, results here could only be applicable to those in later lactation in the current study, although no CP concentration × stage of lactation interactions were found for feed efficiency measures here.

#### Energy use efficiency and methane emissions

Higher DMI as a result of increased DM digestibility in MCP and HCP diets resulted in a higher GEI for these treatments. Subsequently, faecal GE was significantly higher in MCP and HCP groups and HP was highest for HCP compared to the LCP groups. The higher E_L_ for MCP and HCP groups was primarily the result of higher milk yields from these groups. The increased CP intake of MCP and HCP groups resulted in a higher urinary GE output, probably attributed to increased N output often associated with higher CP diets (Katongole and Yan, 2020). Results for urinary GE output are in agreement with Hynes et al. (2016) observing higher urinary GE under diets containing 18.3% CP compared to 16.9 and 17.6% CP (DM basis). Although, other energy input and output variables (GEI, DEI, MEI, faecal GE, CH_4_-E, HP, milk energy, RE) did not differ between diets in Hynes et al. (2016), which is in contrast to the current study. This could be due to the relatively high CP concentrations investigated by Hynes et al. (2016) (16.9–18.3% CP of DM vs 12.2–18.1% CP of DM investigated in the current study) resulting in similar DM digestibility between diets and the subsequent lack of effect of diet on DMI and GEI, since the rate of increase in DM digestibility reduces as CP concentration increases (Roffler et al., 1986, Allen, 2000).

Hynes et al. (2016) observed that a grazing diet containing 17.0% CP of DM would provide sufficient degradable CP for microbial activity and MP synthesis to sustain milk production, energy digestibility, metabolisability and k_L_. The current study is in agreement with this value observing that energy digestibility and metabolisability (DEI/GEI and MEI/GEI) reduced when CP concentration reduced from 18.1 to 15.1% CP of DM, although pairwise comparisons suggested that values for MEI/DEI and E_L(0)_/MEI were the same for 15.1 and 18.1% CP of DM.

Differing CP concentrations did not result in any reduction in overall CH_4_ (g/day) in agreement with Hynes et al. (2016). However, linear reductions in CH_4_ as a proportion of DMI might be associated with higher DMI as CP concentration increases. Yan et al. (2006b) found positive associations between CP concentration and CH_4_ (g/day) and negative associations with CH_4_/DMI and CH_4_/milk. It has been suggested that increased productivity would be a suitable strategy to reduce overall CH_4_ yield and intensity by reducing the number of animals required to produce the same amount of milk over lactation (Yan et al., 2006b). Reducing CH_4_ as a proportion of milk yield or DMI is achieved by increasing DMI in order to increase the rate of passage through the rumen and limit time available for methanogenesis (Yan et al., 2006b, Grandl et al., 2016). Although the current study found no effect of diet on overall CH_4_ production (g/day) or CH_4_/DMI, MCP and HCP diets resulted in less CH_4_ as a proportion of digestible DMI, ECMY and less CH_4_-E (MJ) as a proportion of GEI, DEI and MEI; which may imply that a dietary CP concentration of 12.2% may also have negative implications for CH_4_ parameters in dairy production (beyond its negative effect on production and efficiency).

The current regression equations predict a reduction in CH_4_ as a proportion of digestible DMI or ECMY by 6.1 or 5.3 g/kg, with each incremental increase of CP by 10%. Increasing DMI increases milk yield and simultaneously increases the rate of passage of digesta in the rumen which limits the time available for microbial action thus reducing CH_4_ yield (Yan et al., 2006b). The current findings could potentially be related to the increased DM digestibility and subsequent higher DMI of cows consuming MCP and HCP diets. The lack of significant effects for CH_4_ parameters by Hynes et al. (2016) can be attributed to the significantly higher CP concentrations and subsequent similarity between digestibility and DMI (Yan et al., 2006b). However, the current study found no difference between MCP and HCP groups, suggesting that a dietary CP concentration of 15.0% would be sufficient to increase digestibility and DMI enough to improve productivity and reduce CH_4_ yield and intensity. However, results should be interpreted with caution, as with other substitution studies, diets formulated in this study differ in starch concentration; with increasing CP concentration being coupled with a simultaneous decrease in starch concentration (208, 190 and 170 g/kg DM for LCP, MCP and HCP, respectively). Therefore, the impact of other feed components on variables cannot be ruled out considering that starch is a major driver for DMI (Lechartier and Peyraud, 2010). However, differences in starch concentration between the three diets are relatively small, with the LCP treatment being 1.8% higher than MPC and the MCP being 2.0% higher than the HCP treatment.

### Effect of stage of lactation

Results for liveweight and body condition score are consistent with previous findings observing increasing LW and body condition score as lactation progressed (Yan et al., 2006a, Letelier et al., 2022). This can be attributed to the ability of dairy cows, particularly high genetic merit Holstein-Friesians, to partition more energy into milk production and is often associated with observed negative energy balance during early−lactation (Veerkamp et al., 1994). This was also the case for the current study, observing negative values for RE and the highest milk energy in early−lactation. Intake values are consistent with previous studies reporting decreases in total DMI throughout lactation (Letelier et al., 2022). Although Letelier et al., 2022 found CP concentration × stage of lactation interactions, DMI was highest during early– (86 ± 14.9 days), mid-early (119 ± 10.0 days) and late– (239 ± 11.1 days) lactation when cows were offered diets containing 16.7% CP of DM but mid−late (167 ± 22.2 days) lactation cows had the highest DMI when fed diets containing 18.3% CP of DM. However, the current study found no CP concentration × stage of lactation for pairwise comparison analysis for any variables measured. Similar to Letelier et al. (2022), milk yield and ECMY were lower in late−lactation compared to early and mid-lactation. Milk constituent concentration has been shown to increase throughout lactation as a result of the concentrating effect of decreasing milk yield as lactation progresses (Auldist et al., 1998). However, results here found no difference between mid− and late−lactation for milk fat concentration, similar to Nantapo et al. (2014) and protein and lactose concentrations reduced in late−lactation could be a result of reduced concentrate DMI (Xue et al., 2011) reported in the later stages of lactation.

Stage of lactation had significant impacts on all feed efficiency parameters in the current study in agreement with Letelier et al. (2022) which observed decreases in milk yield/DMI from early to late−lactation, probably a result of a higher rate of reduction in milk yield than DMI. Whilst both CP concentration and stage of lactation had impacts on productivity and feed efficiency variables, there were no CP × stage of lactation interaction for feed efficiency measures suggesting that the effects of CP concentration were not influenced by stage of lactation for these variables.

Xue et al. (2011) found no effect of the stage of lactation on milk energy, contrary to the current study, observing the highest milk energy during early and mid, compared to late−lactation. In their study, MEI was higher during late−lactation (189–266) compared to early (35–112) (Xue et al., 2011). The current study found that MEI was highest during mid−lactation (150–170 days) compared to early (70–90 days) and late (230–250) while, Xue et al. (2011) compared early and late−lactation using four lactation periods and such differences in the diet and days in milk may explain the slight differences between the two studies. Milk energy output/MEI was higher in early−lactation compared to late−lactation in previous work (Xue et al., 2011), in agreement with the present study and is a result of the increased partitioning of energy towards milk production during early−lactation. As lactation progresses, the proportion of ME partitioned towards milk reduces and the proportion partitioned towards body tissue is increased (Xue et al., 2011). This is supported by the findings in the current study observing reductions in E_L_/MEI and increased RE as lactation progresses. Yan et al. (2006b) observed that the ability of cows to partition more ME towards milk production was only achieved during early−lactation under high−concentrate diets. Thus, the high−concentrate diets offered in the current study could have contributed towards higher E_L_/MEI during early−lactation.

Previous studies have found that CH_4_ production increases from early to late−lactation (Garnsworthy et al., 2012, Bielak et al., 2016, Bittante et al., 2018, Lyons et al., 2018) in agreement with the current findings. Lyons et al. (2018) attributes this to differences in bacterial and archaeal communities between early and late−lactation, observing a higher abundance of *Methanobrevibacter* and a lower abundance of *Lactobacillus* and *Arthrobacter* in late−lactation. Methane yield (CH_4_/DMI) and intensity (CH_4_/milk) also increased as lactation progressed. Reductions in CH_4_ as a proportion of DMI or milk yield have been attributed to increased feed intake and improving productivity by means of increasing DMI has been suggested as a suitable strategy to reduce CH_4_ yield and intensity (Yan et al., 2006b). Whilst all CH_4_ production variables measured seemed to increase as lactation progressed, there was no difference in CH_4_ per kg of digestible DMI and ECMY or CH_4_-E (MJ) per GE, DE and ME intakes (MJ) between early and mid-lactation. Lyons et al. (2018) reported that the shift in microbial community was more pronounced between mid− and late−lactation than early to mid and could explain the lack of difference between early and mid−lactation in the current study for some variables.

### Effect of parity

The effect of parity on feed intake was primarily due to multiparous cows being heavier and subsequently having higher DMI (Azizi et al., 2009); this resulted in higher milk yield and ECMY, thus being consistent with previous findings (Morales-Piñeyrúa et al., 2022). There were no effects of parity on feed efficiency parameters. Energy intakes and outputs were all higher for multiparous cows, probably as a result of larger LW and increased DMI (Azizi et al., 2009). Whilst there were no effects of parity on energy use efficiency variables, there were significant CP concentration × parity effects on DEI/GEI, MEI/GEI and E_L_/MEI. Primiparous cows under the LCP treatment had lower DEI/GEI and MEI/GEI than primiparous in MCP and HCP treatments and multiparous cows in the HCP treatment. This may be partly attributable to the higher digestibility of higher CP concentrations diets. Interestingly, primiparous cows in the LCP, HCP and multiparous cows in MCP and HCP treatments were able to partition more dietary MEI towards milk production (higher E_L_/MEI) than multiparous cows in the LCP treatment. Higher E_L_/MEI in the MCP and HCP treatments can be attributed to the higher supply of MP as a result of higher CP feeding (Olmos Colmenero and Broderick, 2006).

The findings of higher total CH_4_ emissions (g/d) for multiparous compared to primiparous cows are consistent with previous investigations (Grandl et al., 2016) and are associated with higher DMI, increased milk yields and higher LW (Grandl et al., 2016). Grandl et al., 2018 also suggested that very young animals and older cows may have a superior digestive energy efficiency with the ability to obtain nutrients without degrading fibre and thus reducing losses as CH_4_. However, animals used in Grandl et al. (2018) ranged from 0.5–12 years of age and a range of their observation would not have been comparable (in terms of parity) to the current study.

There were also stages of lactation × parity interactions identified for a number of productivity variables. Whilst there was a decrease in milk yield across lactation for both primiparous and multiparous cows, primiparous consistently had significantly lower milk yields than multiparous cows, but this difference was not detected in later lactation. The same response was observed for ECMY and E_L_ and could be due to the fact that (i) multiparous cows had significantly higher LW and thus higher DMI enabling them to have superior yields (milk and ECM) compared to primiparous cows (Azizi et al., 2009) and (ii) Holstein cows have a superior ability to divert more energy towards milk production, but mostly during early−lactation and when offered high concentrate diets (Yan et al., 2006a). These factors may explain the similar results between primiparous and multiparous cows for milk yield, ECMY and E_L_ during late−lactation. Gross energy intake was highest for multiparous cows in early and mid−lactation and lowest for primiparous cows in early−lactation. A higher GEI in early−lactation would be expected and results found here are not consistent with previous findings of higher GEI in early−lactation which diminishes as lactation progresses (Xue et al., 2011). However, the present study also found that multiparous cows had numerically higher GEI in mid−lactation, compared with early−lactation, although the difference was not statistically significant. However, the current study investigated three periods of lactation whilst Xue et al. (2011) pooled data from two separate periods for “early” and another two periods for “late” which could explain the differences in DMI and MEI found between the two studies. Urinary GE reflects that of GEI with primiparous cows showing the lowest levels during early−lactation and multiparous cows showing the highest levels in mid−lactation when GEI was also lowest and highest, respectively. Results here are likely to be a result of excess N in urine which is often associated with high CP diets (Hynes et al., 2016).

## Conclusions

Based on results of the present study, whilst the results from residual maximum likelihood analysis showed similar results for milk yield, ECMY and efficiency parameters (ECMY/DMI, milk solids/DMI and milk energy/DMI) between groups of cows fed 151 g/kg or 181 g/kg CP of DM across the whole lactation, the regression analysis illustrated that milk and ECMY could continue to increase between 150 and 180 g/kg DM; but with a peak showing at CP of 170 g/kg DM for milk yield (28.0 kg/d) and at a CP of 180 g/kg DM for ECMY (27.5 kg/d). The optimum CP concentration for milk/DMI was found at 160 g/kg DM. Furthermore, linear interaction between CP concentration × stage of lactation for productivity measures suggests that responses to CP concentration could be dependent on the stage of lactation, with effects on milk yield and ECMY being numerically smaller between CP treatments as lactation progresses. Low diet CP concentrations (122 g/kg) had negative implications for productivity and efficiency, as well as CH_4_ yield and intensity (CH_4_ as a proportion of digestible DMI, ECMY and CH_4_-E, GEI, DEI and MEI). Overall, a diet with CP concentration of between 151 and 181 g/kg DM would be able to provide sufficient protein to maintain milk production and feeding efficiency while reducing CH_4_ as a proportion of feed intake and milk yield. An optimal CP concentration in this study, according to the regression analysis was found at a dietary CP concentration of 170 g/kg and 180 g/kg of dietary CP for ECMY, but these results may also be dependent on the stage of lactation.

## Supplementary material

Supplementary Material for this article (https://doi.org/10.1016/j.animal.2025.101426) can be found at the foot of the online page, in the Appendix section.

## Ethics approval

All procedures adopted in the present experiments were approved by the Ethical Review Committee of the Agri-Food and Biosciences Institute (Hillsborough, UK) under the Project Licence of 2587b and were in accordance with the UK Animal Scientific Procedures Act (1986).

## Data and model availability statement

The dataset supporting the conclusions of this article is not deposited in an official repository but is available on request from the corresponding authors.

## Declaration of Generative AI and AI-assisted technologies in the writing process

During the preparation of this work the author(s) did not use any AI and AI-assisted technologies.

## Author ORCIDs

**S.O:**https://orcid.org/0000-0003-1012-3891.

**T.Y:**https://orcid.org/0000-0002-1994-5202.

**X.C:**https://orcid.org/0000-0002-9056-578X.

**A.W.G.:**https://orcid.org/0000-0002-9806-6424.

**K.T:**https://orcid.org/0000-0002-2848-5594.

**S.H:**https://orcid.org/0000-0002-9284-2453.

**S.S:**https://orcid.org/0000-0002-7293-182X.

## CRediT authorship contribution statement

**S. Ormston:** Validation, Methodology, Investigation, Formal analysis, Data curation, Conceptualisation. **T. Yan:** Writing – review & editing, Writing – original draft, Visualisation, Validation, Supervision, Resources, Project administration, Methodology, Investigation, Funding acquisition, Data curation, Conceptualisation. **X. Chen:** Writing – review & editing, Methodology, Investigation, Data curation. **A.W. Gordon:** Writing – review & editing, Software, Methodology, Formal analysis. **K. Theodoridou:** Writing – review & editing, Supervision. **S. Huws:** Writing – review & editing, Supervision. **S. Stergiadis:** Writing – review & editing, Writing – original draft, Visualisation, Project administration, Methodology, Funding acquisition, Data curation, Conceptualisation.

## Declaration of interest

None.
