# Supplementary material for: Efficiency of feed and energy use in primiparous and multiparous dairy cows fed contrasting dietary protein concentrations across lactation
Source: Animal. 2025 Mar;19(3):101426. doi: 10.1016/j.animal.2025.101426 (PMC11904127; doi:10.1016/j.animal.2025.101426)
Supplement: Supplementary Data 1 [file mmc1.pdf]

Animal

Efficiency of feed and energy use in primiparous and multiparous dairy cows fed contrasting dietary protein concentrations across lactation

Sabrina Ormston[1], Tianhai Yan[2], Xianjiang Chen[2], Alan W. Gordon[3], Katerina Theodoridou[4], Sharon Huws[4], Sokratis Stergiadis[1]*

[1]*Department of Animal Sciences, School of Agriculture, Policy and Development, University of Reading, PO Box 237, Earley Gate, Reading, RG6 6EU, United Kingdom*

[2]*Agri-Food and Biosciences Institute, Hillsborough, Co. Down, BT26 6DR, United Kingdom*

[3] *Agri-Food and Biosciences Institute, Statistical Services Branch, Newforge Lane, Belfast, Co. Antrim, BT9 5PX, United Kingdom*

[4]*Institute for Global Food Security, Queen's University Belfast, Belfast, BT9 5DL, United Kingdom*

*Corresponding author. s.stergiadis@reading.ac.uk (Sokratis Stergiadis)

Supplementary material S1

Materials and methods

Experimental design, animal and diets The 24 cows used in the present experiment comprised a sub-group of the cows involved in a whole lactation study (Law et al., 2009). The whole lactation study utilized 90 autumn calving Holstein-Friesian (**HF**) dairy cows (45 primiparous and 45 multiparous with a mean parity of 3.1) which were allocated to a total mixed ration (**TMR**) containing 1 of 3 dietary CP concentrations (Target concentrations were 120, 150 and 180 g CP/kg DM: Low CP, Medium CP, and High CP, respectively. Actual concentrations were 114, 144 and 173 g CP/kg DM) from calving until day 150 of lactation. At day 151 of lactation half the animals in each treatment were offered a diet with alternative dietary CP concentrations. Cows were balanced for parity, calving date, milk yield and BW. Of these cows, those receiving 114 g CP/kg DM went onto 144 g CP/kg DM, 144 g CP/kg DM went onto 173 g CP/kg DM and 173 g CP/kg DM went onto 144 g CP/kg DM diets. The remaining animals continued their original diets. Primiparous animals were assigned in a balanced manner to treatments based on heifer rearing regime, calving date and live weight. Multiparous animals were assigned to treatments according to parity, previous lactation milk yield, calving date and live weight. In the current study, 24 cows (8 cows from each treatment) remained on their original diet until day 305 of lactation. At early, mid and late lactations (70-80, 150-160 and 230-240 days) all 8 cows from each treatment were transferred to metabolism units for total diet digestibility and nutrient utilization measurements. The TMR offered contained (g/kg DM) 270 grass silage, 180 maize silage and 550 concentrates. The target dietary CP concentrations were achieved by manipulating proportions of 2 concentrate meals containing either high or low concentration of CP. Dietary ingredients and chemical compositions of the high and low CP concentrates are outlined in Table S1. The two concentrates contained the

same level of metabolizable energy (**ME**) (13.1 MJ/kg DM), but different concentrations of CP (229 vs. 117 g/kg DM). These variables were calculated using the UK Feed into Milk program (Feed into Thomas, 2004). Actual mean CP concentrations for this continuous study were 122, 151 and 181 g CP/kg DM (LCP, MCP and HCP, respectively). Cows were milked twice daily starting at 0500 and 1630 h, respectively, had *ad libitum* access to fresh water and TMR which was delivered to animals between 1000 and 1100 h daily. Digestibility study and sample measurements throughout the study, from calving to day 305 of lactation, total feed intake was recorded daily. For digestibility measurements, animals were transferred to metabolism units and housed in individual stalls for 8 days. Feed intake and outputs of faeces and urine were measured during the final 6 days. Animals were then immediately transferred to indirect open-circuit respiration calorimeter chambers for 3 days with gaseous exchange (oxygen, carbon dioxide and methane) measured during the final 2 days. Grass silage and maize silage were sampled daily during the digestibility period, for determination of fermentation variables (pH, volatile fatty acids, ammonia N, lactic acid, ethanol and propanol concentrations) and N and gross energy concentrations, and oven DM concentration (60°C for 48 h). These oven-dried silage samples were then bulked over the measurement period for determination of ash, neutral detergent fibre (**NDF**) and acid detergent fibre (**ADF)** concentrations. Fresh samples of silage were taken twice weekly for determination of toluene-alcohol-corrected DM, pH, CP, ammonia-N, gross energy (**GE**), lactic acid, volatile fatty acids, ethanol and propanol concentrations. Concentrate samples were taken three days each week during the production study and daily during the digestibility study and bulked weekly for analysis of oven DM, GE, CP, ADF, NDF and ash concentrations. Measurements for faeces and urine outputs were recorded daily along with samples

taken as a proportion (5%) of total excretion of faeces (as weight) and urine (as volume). Daily samples for faeces and urine for each cow were then mixed separately and a representative sample taken for analysis of; fresh faeces samples for oven DM (85°C for 48 h), gross energy and N concentrations, and urine samples for gross energy and N concentrations. Silage DM concentrations were calculated from oven DM concentrations (60°C for 48 h) corrected for the loss of volatile fatty acids, lactic acid, alcohol and ammonia using the equation recommended by Porter and Murray, 2001: corrected DM content (g/kg) = 19.96 + 0.9793 × (0.987× oven DM content (g/kg) − 0.26). Silage ammonia was assayed by bringing the alkalinity of the aqueous extract to pH 10 and using an Orion 95-12-00 ammonia sensing gas electrode. The volatile fatty acids, lactic acid, ethanol and propanol concentrations in silage samples were analysed by gas liquid chromatography using a Perkin-Elmer gas chromatograph (Perkin-Elmer, Waltham, Massachusetts, USA) having a column packed with matrix 80/120 Carbopack B-DA and 4% Carbowax 20M. Ash concentration in silage, concentrate and faeces samples was determined by incineration in a muffle furnace (Vecstar, Derbyshire, UK) at 550°C for approximately 10 h (AOAC, 1990). The N concentrations of fresh silage, concentrate, fresh faeces and urine samples were determined using a Tecator Kjeldahl Auto 1030 Analyzer (Foss Tecator AB, Höganäs, Sweden). Gross energy concentrations in silage, concentrate, faeces and urine samples were measured in an isoperibol bomb calorimeter (Parr Instruments Co., Moline, Illinois). A Fibertec fibre analyser (Foss Electric, Hillerød, Denmark) determined NDF and ADF concentrations in silage and concentrate samples. The NDF was assayed using sodium sulphite and α-amylase, as described by Van Soest et al., 1991.

As also described in Law et al. (2009), throughout the experiment, milk yields were recorded with samples being taken daily during the digestibility and chamber measurements and every second week for 3 days, during the production study.. Samples were then bulked weekly and analysed for fat, N, lactose and energy concentrations. Fat and lactose concentrations were analysed using a Milkoscan Model 605 (Foss Electric, DK-3400, Hillorod, Denmark) using the method of Ling, 1963. Nitrogen and energy concentrations were determined using a Tecator Kjeldahl Auto 1030 Analyzer (Foss Tecator AB, Höganäs, Sweden) and gross energy concentrations were measured in an isoperibol bomb calorimeter (Parr Instruments Co., Moline, Illinois).

Animals were weighed on the first and last day of digestibility measurements, before morning feeding and body condition score (BCS) was determined, with 1 being very thin and 5 very fat as described by Edmonson et al., 1989.

On day nine, cows were transferred to indirect open-circuit respiration calorimeter chambers for a further three days with gaseous exchange (O_2, CO_2 and CH_4) measured during the final two days, following one day of adaptation to the chamber. Recovery rates were 97-103%. Calibration of the chambers was carried out as follows: The analysers are calibrated with gases produced from individual pure analytical standard gases (CH_4, CO_2, O_2) using Wostoff Mixing pumps (Wösthoff Messtechnik, Bochum Germany). This determines the absolute range (0-500 ppm for CH_4) and the linearity within this range. The analysers are calibrated using O_2 free N and the known concentration of a gas (span gas), before each run and is checked every 6 hours. Flow measurement systems are checked with analytical grade CO_2 and N using CO_2 and O_2 analysers by determining the recovery of CO_2 and depletion of O_2.

Statistical analysis

All data points were included in the analysis. A linear mixed model with repeated measures design (residual maximum likelihood analysis; **REML**) was used to investigate the effect of CP level, stage of lactation, parity and all their interactions on productivity, energy and feed use efficiencies and CH_4 parameters. The statistical programme used was GenStat® 18 (VSN International 2020). The fixed effects were CP level (low, 122; medium, 151; and high, 181; g CP/kg DM), stage of lactation (early, 70-80 days; mid, 150-160 days; late, 230-240 days), parity (primiparous; multiparous), and their interactions (CP level, CP level × stage of lactation, CP level × parity, stage of lactation × parity and CP level × stage of lactation × parity). Cow was fitted as the subject factor and stage of lactation as the time factor. Correlation between time points was modelled using an autoregressive model of order one. The adequacy of the models fitted (normality and homogeneity of variance of the residuals) was assessed visually by inspection of the appropriate residual plots. As these indicated no deviation from normality, all variables were analysed untransformed. When any fixed effect was significant for a measured variable ($P < 0.05$), pairwise comparisons of means were performed using Fisher's Least Significant Difference test. Descriptive statistics to generate means and standard errors for presentation in tables and figures, were carried out in Minitab® 20.2. In addition, linear and quadratic effects were also assessed for the main fixed effect diet CP level and its interactions with parity and stage of lactation (CP level, CP level × stage of lactation, CP level × parity and CP level × stage of lactation × parity). Regression equations between diet CP content and variables that were significantly affected by CP content, were developed with CP content (kg/kg DM) being the explanatory variable and by using REML so that the potential random effects of cow, stage of lactation and parity could be accounted for.

Both linear and quadratic regressions were tested. If quadratic effect was statistically significant, the quadratic relationship is presented; or else the linear relationship is presented.

```
FCONTRASTS   [FORM=CP_level*Period*Parity;   NEW=fpol]   CP_level;   ORD=2;

XCON=!(18,15,12); ORTH=Y; DEV=N


FOR DUM=Milk_yield_kg_d
  VCOMPONENTS [FIXED=CP_level*Period*Parity; FACTORIAL=9]
RANDOM=Cow_no.Period;\
   CONSTRAINTS=positive
  VSTRUCTURE [TERMS=Cow_no.Period] FACTOR=Period; MODEL=ar; ORDER=1;
HETEROGENEITY=none
  REML [PRINT=model,components,means,waldTests; MAXCYCLE=30;
FMETHOD=automatic; PSE=differences;\
   MVINCLUDE=explanatory,yvariate; METHOD=AI] DUM
  VAIC [PRINT=aic,sic; INCLUDE=*]
  VMCOMPARISON [PR=LET; METH=FPLSD; DFG=200; DFM=T] CP_level*Period*Parity
  VPLOT [RMETHOD=all] fittedvalues,normal,halfnormal,histogram
  VCOMPONENTS [FIXED=#fpol; FACTORIAL=9] RANDOM=Cow_no.Period;
CONSTRAINTS=positive
  VSTRUCTURE [TERMS=Cow_no.Period] FACTOR=Period; MODEL=ar; ORDER=1;
HETEROGENEITY=none
  REML [PRINT=model,components,waldTests; MAXCYCLE=30; FMETHOD=automatic;
PSE=differences;\
   MVINCLUDE=explanatory,yvariate; METHOD=AI] DUM
ENDFOR
```

Regression equations between diet CP content and variables that were significantly affected by CP content, were developed with CP content (kg/kg DM) being the explanatory variable and by using REML so that the potential random effects of cow, stage of lactation and parity could be accounted for. Both linear and quadratic regressions were tested. If quadratic effect was statistically significant, the quadratic relationship is presented; or else the linear relationship is presented.

```
DELETE [REDEFINE=yes] _remlsave

VCOMPONENTS [FIXED=CP_content_kg_kg*CP_content_kg_kg; FACTORIAL=9;
CADJUST=none]\

RANDOM=Cow_no,Period,Parity;\

INITIAL=1,1,1; CONSTRAINTS=positive,positive,positive

REML [PRINT=model,components,effects,waldTests; PSE=differences;
FMETHOD=automatic; WEIGHTS=*; MVINCLUDE=*; METHOD=AI; MAXCYCLE=20]
Milk_yield_kg_d; SAVE=_remlsave
```

```
VAIC[PRINT=AIC,SIC,DEVIANCE,DFFIXED,DFRANDOM,CHANGES]

VKEEP [FITTEDVALUES=fit; RMETHOD=final]

PRINT CORRELATION (Milk_yield_kg_d;fit)**2
```

Table S1: REML analysis, linear and quadratic responses for the two-way and three-way interactions of CP level, stage of lactation and parity for feed intake, productivity and feed efficiency parameters of multiparous and primiparous cows offered low, medium or high CP diets, across lactation.

	CP × SL			Parity × CP			Parity × CP × SL			Parity × SL
	P-value[1]	P-value[1]		P-value[1]	P-value[1]		P-value[1]	P-value[1]		P-value[1]
		L[2]	Q[2]		L[2]	Q[2]		L[2]	Q[2]	
Feed intake and liveweight										
Liveweight (kg)	0.092	0.241	0.559	0.007	0.004	0.148	0.257	0.087	0.849	0.535
BCS	0.459	0.291	0.566	0.043	0.141	0.036	0.199	0.190	0.252	0.070
Total DM intake (kg/d)	0.146	0.041	0.865	0.146	0.419	0.570	0.160	0.234	0.152	0.006
Grass silage DMI (kg/d)	0.681	0.411	0.785	0.681	0.510	0.863	0.450	0.427	0.372	0.098
Maize silage DMI (kg/d)	0.292	0.098	0.907	0.741	0.474	0.784	0.176	0.081	0.518	0.003
Concentrate DMI (kg/d)	0.165	0.048	0.860	0.593	0.464	0.481	0.309	0.483	0.189	0.030
CP intake (g/d)	0.091	0.028	0.684	0.685	0.662	0.457	0.045	0.045	0.144	0.808
DM digestibility (%)	0.141	0.037	0.935	0.039	0.268	0.020	0.944	0.912	0.758	0.585
Milk production and composition										
Milk Yield (kg/d)	0.082	0.020	0.871	0.727	0.560	0.592	0.267	0.157	0.469	0.039
ECMY (kg/d)	0.138	0.040	0.807	0.340	0.237	0.385	0.310	0.180	0.508	0.041
Milk fat (g/kg)	0.429	0.331	0.445	0.885	0.676	0.801	0.947	0.492	0.782	0.509
Milk protein (g/kg)	0.845	0.775	0.664	0.833	0.597	0.775	0.969	0.894	0.857	0.450
Milk lactose (g/kg)	0.984	0.839	0.989	0.799	0.587	0.700	0.839	0.954	0.521	0.161
Milk fat yield (kg/d)	0.848	0.553	0.920	0.275	0.177	0.384	0.579	0.344	0.676	0.123
Milk protein yield (kg/d)	0.216	0.100	0.555	0.878	0.947	0.614	0.852	0.613	0.828	0.267
Milk lactose yield (kg/d)	0.092	0.190	0.975	0.725	0.871	0.472	0.108	0.396	0.365	0.003
Feed efficiency parameters										
Residual feed intake (kg/day)[3]	0.927	0.656	0.994	0.404	0.787	0.192	0.181	0.064	0.707	0.232

Milk/DMI	0.554	0.317	0.708	0.496	0.440	0.373	0.579	0.271	0.905	0.548
ECMY/DMI	0.730	0.547	0.671	0.076	0.088	0.120	0.790	0.449	0.968	0.841
Milk solids/DMI[4]	0.802	0.601	0.742	0.195	0.267	0.150	0.799	0.472	0.944	0.540
Milk energy (E_L)/DMI (MJ/DMI kg)	0.730	0.547	0.671	0.076	0.088	0.120	0.790	0.449	0.968	0.841
Milk/concentrate DMI (kg/kg)	0.609	0.402	0.654	0.446	0.422	0.327	0.517	0.208	0.984	0.618
Milk fat/DMI (g/kg)	0.954	0.782	0.917	0.055	0.049	0.148	0.947	0.722	0.967	0.951
Milk fat/concentrate DMI (g/kg)	0.945	0.760	0.913	0.044	0.047	0.111	0.896	0.628	0.934	0.879
Milk protein/DMI (g/kg)	0.410	0.314	0.438	0.712	0.820	0.433	0.984	0.852	0.971	0.744
Milk protein/concentrate DMI (g/kg)	0.481	0.392	0.450	0.674	0.810	0.397	0.967	0.804	0.946	0.727
Milk/CP DMI (kg/kg)	0.965	0.832	0.904	0.372	0.381	0.272	0.728	0.407	0.902	0.528

Abbreviations: REML = residual maximum likelihood analysis, SL = stage of lactation, BCS = body condition score; DMI = dry matter intake; ECMY = energy corrected milk yield.

CP concentrations for LCP, MCP and HCP were 122, 151 and 181 g/kg DM, respectively.

Early = 70-90 days; Mid = 150-170 days; Late = 230 -250 days.

[1]Significances were declared at $P < 0.05$.

[2]Significance for Linear (L) and Quadratic (Q) effects.

[3]Residual feed intake = actual DMI (kg/d) – predicted DM requirement (kg/d).

[4]Milk solids calculated as total fat, protein and lactose (g/d).

Table S2. REML analysis, linear and quadratic responses for the two-way and three-way interactions of CP, stage of lactation and parity for energy intake and output, EUE parameters and CH_4 production parameters of multiparous and primiparous cows offered low, medium or high CP diets, across lactation.

	CP × SL			Parity × CP			Parity × CP × SL			Parity × SL
	P-value[1]	P-value[1]		P-value[1]	P-value[1]		P-value[1]	P-value[1]		P-value[1]
		L[2]	Q[2]		L[2]	Q[2]		L[2]	Q[2]	
Energy parameters (MJ/d)										
GEI	0.127	0.033	0.904	0.665	0.445	0.639	0.124	0.157	0.159	0.007
DEI	0.072	0.018	0.825	0.220	0.292	0.163	0.087	0.129	0.118	0.008
MEI	0.085	0.022	0.795	0.252	0.232	0.244	0.101	0.126	0.150	0.006
Faecal GE	0.240	0.072	0.953	0.494	0.938	0.241	0.861	0.717	0.735	0.215
Urinary GE	0.085	0.022	0.795	0.252	0.232	0.244	0.101	0.126	0.150	0.006
CH_4-E	0.103	0.094	0.208	0.163	0.805	0.062	0.484	0.794	0.229	0.335
HP[2]	0.595	0.300	0.852	0.101	0.196	0.082	0.295	0.923	0.098	0.160
E_L	0.138	0.040	0.807	0.340	0.237	0.385	0.310	0.180	0.508	0.041
RE	0.955	0.830	0.867	0.254	0.149	0.415	0.656	0.311	0.980	0.244
Energy use efficiency										
DEI/GEI	0.200	0.059	0.911	0.045	0.303	0.023	0.897	0.869	0.676	0.619
MEI/GEI	0.314	0.128	0.749	0.033	0.080	0.043	0.880	0.773	0.722	0.331
MEI/DEI	0.560	0.564	0.402	0.213	0.149	0.308	0.981	0.836	0.972	0.107
HP/MEI[3]	0.764	0.413	0.983	0.771	0.543	0.707	0.611	0.275	0.977	0.068
E_L/MEI	0.974	0.969	0.810	0.011	0.025	0.029	0.808	0.541	0.842	0.942
$E_{L(0)}$/MEI	0.838	0.504	0.986	0.728	0.713	0.484	0.463	0.180	0.960	0.064
K_L	0.926	0.715	0.906	0.482	0.416	0.374	0.134	0.071	0.409	0.309
Methane parameters										
CH_4 (g/d)	0.106	0.093	0.223	0.175	0.837	0.066	0.521	0.809	0.253	0.295
CH_4/DMI (g/kg)	0.480	0.411	0.427	0.240	0.370	0.151	0.982	0.868	0.945	0.163
CH_4/DDMI (g/kg)	0.738	0.784	0.479	0.273	0.222	0.291	0.980	0.837	0.971	0.116

CH_4/Milk yield (g/kg)	0.278	0.561	0.141	0.378	0.695	0.185	0.689	0.356	0.931	0.129
CH_4/ECMY (g/kg)	0.210	0.505	0.106	0.160	0.511	0.073	0.639	0.312	0.929	0.139
CH_4-E /GEI (MJ/MJ)	0.384	0.349	0.356	0.210	0.346	0.133	0.978	0.892	0.901	0.177
CH_4-E /DEI (MJ/MJ)	0.533	0.584	0.358	0.228	0.194	0.255	0.979	0.853	0.944	0.134
CH_4-E /MEI (MJ/MJ)	0.565	0.609	0.379	0.227	0.187	0.264	0.981	0.858	0.950	0.133

Abbreviations: REML = residual maximum likelihood analysis, EUE = energy use efficiency, SL = stage of lactation, GEI = gross energy intake, GE = gross energy, HP = heat production, DEI = digestible energy intake, MEI = metabolizable energy intake, E_L = milk energy output (MJ/d), $E_{L(0)}$ = E_L adjusted to 0 energy balance, K_L = efficiency of utilisation of ME for milk production CH_4 = methane; DMI = dry matter intake; DDMI = digestible DMI, ECMY = energy corrected milk yield, CH_4-E = CH_4 energy.

CP concentrations for LCP, MCP and HCP were 122, 151 and 181 g/kg DM, respectively.

Early = 70-90 days; Mid = 150-170 days; Late = 230 -250 days.

[1]Significances were declared at P <0.05.

[2]Significance for Linear (L) and Quadratic (Q) effects.

[3]Heat production was calculated based on O_2 consumption, CO_2 and CH_4 production and urinary N excretion using the equation of Brouwer, 1965; $[(16.18 \times O_2) + (5.16 \times CO_2) - (2.42 \times CH_4) - (5.9 \times UN)]/1\,000$.

References

Brouwer, E., 1965. Report of Sub-Committee on Constants and Factors. In: Proceedings of the 3rd Symposium on Energy Metabolism (ed. Blaxter, K.L.). Academic Press, London, UK, pp. 441-443.

Edmonson, A.J., Lean, I.J., Weaver, L.D., Farver, T., Webster, G., 1989. A Body Condition Scoring Chart for Holstein Dairy Cows. Journal of Dairy Science 72, 68-78. doi:https://doi.org/10.3168/jds.S0022-0302(89)79081-0.

Law, R.A., Young, F.J., Patterson, D.C., Kilpatrick, D.J., Wylie, A.R., Mayne, C.S., 2009. Effect of dietary protein content on animal production and blood metabolites of dairy cows during lactation. Journal of Dairy Science 92, 1001-1012. doi:http://10.3168/jds.2008-1155.

Ling, E.R., 1963. A textbook of Dairy Chemistry: Practical, 3rd. Chapman and Hall Ltd, London, UK.

Porter, M.G., Murray, R.S., 2001. The volatility of components of grass silage on oven drying and the inter-relationship between dry-matter content estimated by different analytical methods. Grass and Forage Science 56, 405-411. doi:https://doi.org/10.1046/j.1365-2494.2001.00292.x.

Thomas, C., 2004. Feed into Milk: An advisory manual. Nottingham University Press, Nottingham, UK.

Van Soest, P.J., Robertson, J.B., Lewis, B.A., 1991. Methods for Dietary Fiber, Neutral Detergent Fiber, and Nonstarch Polysaccharides in Relation to Animal Nutrition. Journal of Dairy Science 74, 3583-3597. doi:https://doi.org/10.3168/jds.S0022-0302(91)78551-2.

VSN International 2020. Genstat for Windows, 18th edition. CSN International, Hempstead, UK.
